# Cracking ERα Y537S Resistance: Explainable Machine Learning-Guided Discovery and Molecular Dynamics Validation of Stable Candidate Ligands

**DOI:** 10.3390/ph19091471

**Published:** 2026-09-16

**Authors:** Abdulmohsen M. Alruwetei

**Affiliations:** Department of Medical Laboratories, College of Applied Medical Sciences, Qassim University, Buraydah 51431, Saudi Arabia; roietaie@qu.edu.sa

**Keywords:** estrogen receptor alpha, endocrine resistance, Y537S mutation, machine learning, MD simulation

## Abstract

**Background/Objectives:** Endocrine resistance due to activating mutations in the estrogen receptor alpha (ERα), especially Y537S, is still a big challenge in the treatment of hormone receptor-positive breast cancer, and there is a need to develop new small-molecule inhibitors that can overcome ligand-independent receptor activation. We used an integrated machine learning and molecular dynamics (MD)-based virtual screening (VS) approach to identify potential computationally prioritized hits of ERα Y537S in this study. **Methods:** The dataset (~3353 compounds) was characterized using molecular fingerprints and graphically represented by PCA and t-SNE to show structurally different clusters linked to potency (pIC50). To guarantee robust downstream model training, the diagnosis of structural and influential outliers was completed through the application of rigorous data quality control methods, including the Williams plot, Mahalanobis distance, and Cook’s distance diagnostics. This was followed by multiple benchmarks of machine learning classifiers (random forest, SVM, KNN, and CNN) for activity classification, yielding the highest AUC of 0.95 for the random forest classifier. Interpretable structure–activity insights were gained by the identification of key substructures of the fingerprint by SHAP and feature importance analysis that influence the predicted potency. Three lead candidates (Hit-1, Hit-2, and Hit-3) were screened and tested with a 200 ns all-atom MD simulation in comparison with a reference control to evaluate the binding stability. Comprehensive trajectory analysis, such as PCA, FEL, RDF, salt bridges, and DCCM, was completed following the structure-based identification of the most stable ligands. **Results:** Three candidates were identified as Hit-1, Hit-2, and Hit-3. Hit-3 was found to be bound in the most stable binding pose and had the most similar conformational behavior to the control, whereas Hit-2 showed transient pose instability associated with increased anti-correlated domain-level motions and an alternate high-salt-bridge conformational state. As no computational metric can be an absolute measure of experimental affinity, Hit-1 has the most favorable calculated end-point binding-energy estimate, while Hit-3 showed conformational stability in MD simulations. **Conclusions:** The computationally prioritized candidates can be subjected to further experimental testing, as the conducted docking, MD simulations, and end-point free energy analyses cannot establish overall compound binding, cell-based activity, pharmacological mechanism, or therapeutic efficacy.

## 1. Introduction

Estrogen receptor alpha (ERα) signaling is involved in the majority of breast cancers diagnosed, and breast cancer remains one of the most common malignancies among women worldwide. ERα is a nuclear transcription factor that, after binding to estrogen, undergoes conformational changes that allow recruitment of coactivators and induction of transcription of genes responsible for cell proliferation and survival. This estrogen dependency has made ERα the foundation for endocrine therapies such as selective estrogen receptor modulators (tamoxifen), aromatase inhibitors, and selective estrogen receptor degraders (fulvestrant), which have greatly improved the outcomes of ER-positive breast cancer patients. Although effective in the beginning, acquired resistance is a significant clinical problem, especially in the long-term and metastatic endocrine-treated setting [1]. One of the major factors that plays a role in this resistance is the formation of point mutations in the ERα LBD (most commonly at Y537). In particular, the Y537S mutation is associated with a constitutively active conformation of the receptor that binds to the coactivator (e.g., GRIP/SRC family) and results in transcriptional activity without the presence of estrogen. The Y537S mutation is a ligand-independent activation mutation that is responsible for aromatase inhibitor resistance and decreased sensitivity to multiple antiestrogens, making it an important target for designing new ER-targeted therapeutics [2]. A combination of modifiable and non-modifiable factors shapes breast cancer risk. Non-modifiable risk factors are inherent genetic mutations or reproductive or hormonal histories that influence lifetime estrogen exposure, while modifiable risk factors are excess body weight, smoking, alcohol consumption, and use of hormone-based medications [3]. Breast cancer is heterogeneous, and the tumors vary in molecular characteristics, biological behavior, and clinical prognosis. The diversity was elucidated by investigations into gene expression, which resulted in intrinsic molecular subtypes being defined and ultimately led to the current molecular classification systems [4]. Breast cancers are therefore typically classified into four major groups: Luminal A, Luminal B, HER2-enriched, and triple-negative breast cancer (TNBC), which lacks expression of the estrogen receptor (ER), progesterone receptor (PR), and HER2 and lacks some proliferation markers [5]. Of breast cancers, around 70% are ER-positive, according to the standard immunohistochemistry procedure; ER positivity is now defined as >1% of the tumor cells with nuclear staining [6]. The fact that these features highlight the pivotal role of ERα in ER-positive disease provides a strong drive to investigate the interplay between ER and immune regulation. The ESR1 gene, which encodes ERα, is located on chromosome 6q25 and belongs to the family of nuclear receptors, which are ligand-activated transcription factors [7]. The receptor consists of 595 amino acids and is involved in various physiological functions such as reproduction, development, metabolism, and embryogenesis. ERα activation promotes the transcription of many genes, such as MYC, CCND1, FOXM1, GREB1, BCL2, and IGF1. Many of these targets have oncogenic activity and are involved in cell proliferation, survival, sensitivity to DNA-damaging agents, etc., which are involved in estrogen-dependent growth [8]. Under steady-state conditions, ERα is predominantly nuclear, where it can regulate estrogen-responsive genes efficiently, but it can also enter the cytoplasm [9]. Despite significant progress in understanding the structural basis of endocrine resistance in breast cancer, therapeutic targeting of the ERα Y537S ligand-binding domain mutant remains a clinical challenge, with the conventional antiestrogens, tamoxifen and fulvestrant, having significantly lower efficacy against this constitutively active variant. The mutation is specifically related to endocrine therapy resistance in ER-positive breast cancer. The mutation causes Tyr537 to be replaced with Serine. It is located in helix 12 [10]. Current computational efforts have been aimed at either the wild-type ERα or have been based on a single method (either docking or dynamics) without incorporating predictive machine learning models to prioritize compounds before structural evaluation. In the present study, a multi-tiered computational strategy is employed, including machine learning-based bioactivity prediction, molecular docking of the 6CBZ crystal structure, and molecular dynamics simulation, to systematically discover and elucidate novel small molecules that could disrupt the aberrant ERα Y537S signaling axis, providing a more robust and mechanistically sound framework for lead identification for the treatment of endocrine-resistant breast cancer.

## 2. Results

### 2.1. QSAR Model Development and Validation

A comprehensive QSAR and machine learning workflow was adopted to investigate the structural requirements for biological activity and build a predictive model. The chemical diversity of the compound set was first explored using dimensionality reduction methods and then by the applicability domain assessment to determine the reliability of the compound set. In order to identify which modelling methods are more interpretable, SHAP-based feature importance analysis was conducted, and the predictive ability of several machine learning methods was compared to determine the best predictive model for pIC50 prediction.

#### 2.1.1. Chemical Space Analysis of Molecular Fingerprints

**Figure 1:**
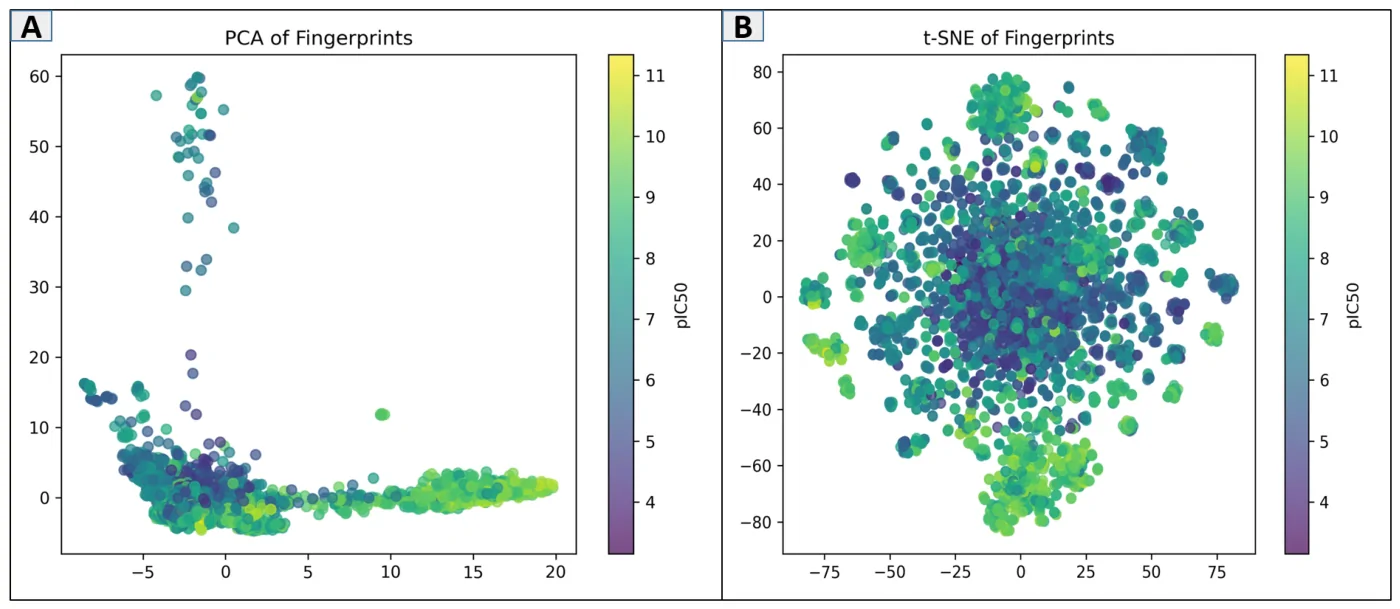
Dimensionality reduction in molecular fingerprints colored by the pIC50 value of the compound. Chemical space projections showing the distribution and clustering of compounds based on their bioactivity (pIC50): (**A**) principal component analysis (PCA) and (**B**) t-distributed stochastic neighbor embedding (t-SNE).

The molecular space of the compound library was visualized using PCA (Figure 1A) and t-SNE (Figure 1B) to reduce the dimensionality of the molecular fingerprints and to relate the chemical space to potency in terms of the pIC50 value. Using t-SNE, which maintains the local region structure, multiple distinct clusters of structurally similar compounds were found, with several of these clusters exhibiting some relative homogeneity in pIC50 values, suggesting a local structure–activity relationship within that cluster [11].

#### 2.1.2. Applicability Domain Analysis

**Figure 2:**
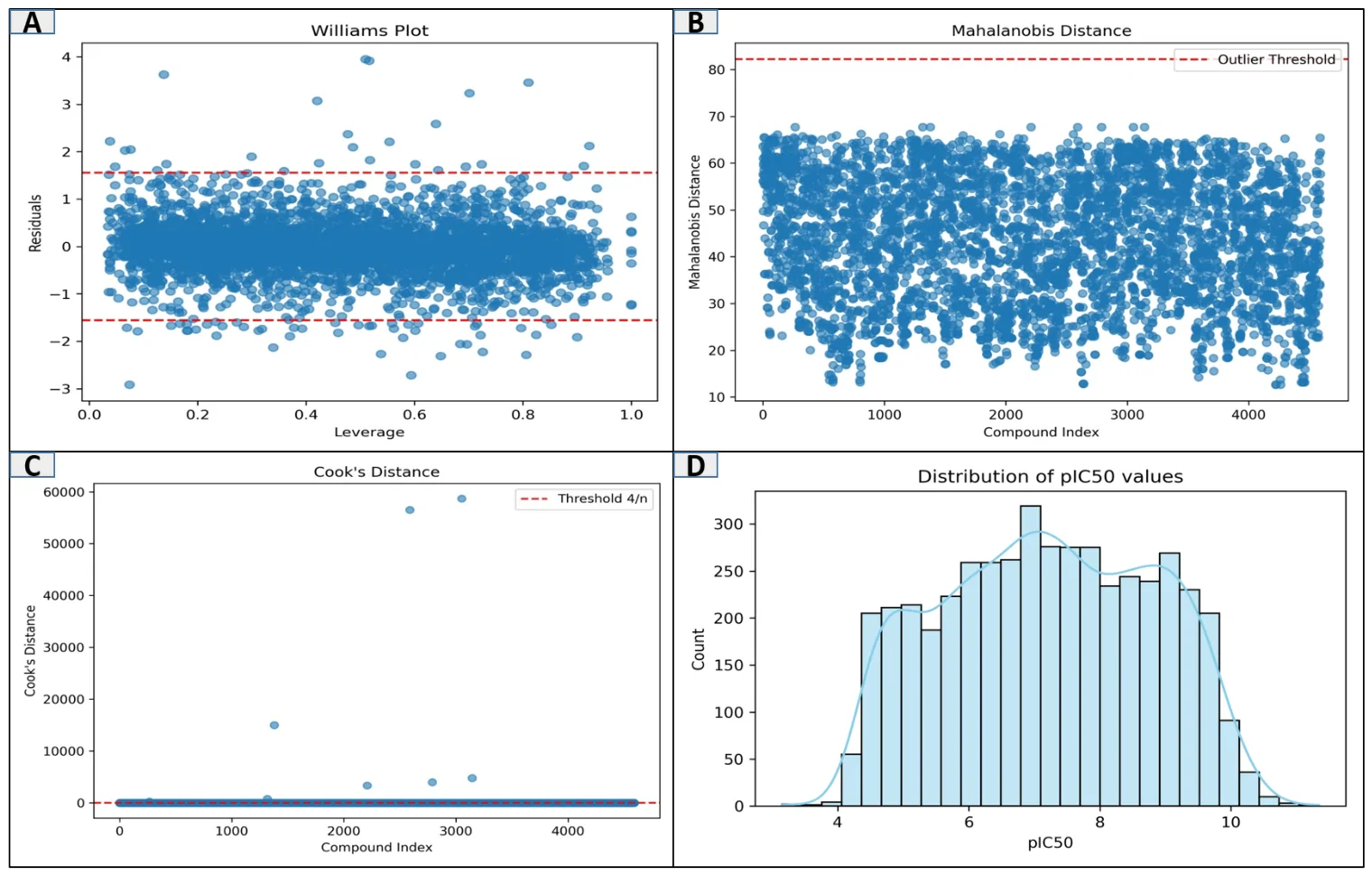
Statistical diagnostics on the compound dataset were employed to develop a model. (**A**). Williams plot for structural outliers and response outliers to the applicability domain. Each compound in the dataset has a distance from the dataset centroid, denoted as (**B**), with the dashed line representing the outlier limit. (**C**) Distance from Cook for each compound index, with influential points highlighted with respect to the threshold (4/*n*). (**D**) Distribution of pIC50 values for the set, with overlaid density curve for the overall spread of bioactivity.

An applicability domain (AD) assessment and model diagnostics were conducted to assess the reliability of the QSAR predictions and also to describe the chemical space covered by the training set [12]. The reliable prediction region was identified from the Williams plot (Figure 2A) and leverage values and standardized residuals. Leverage and residual limits were within predefined limits, with a few response outliers noted. No compound had high leverage with large residuals, meaning that most of the compounds modeled were inside the reliable domain of the QSAR. To find compounds that were far removed from the multivariate descriptor space of the training set, Mahalanobis distance analysis was also performed (Figure 2B). The MAU of the modeled chemical space was not assessed for significant multivariate outliers. Individual observation effects on model fitting were evaluated using Cook’s distance analysis (Figure 2C). About five compounds had a relatively strong influence and were studied in this sensitivity analysis. These observations were kept because they would not significantly impact the overall model performance. For the virtual screening compounds, the applicability domain assessment was then taken into account for interpretation of the ML/QSAR predictions. Predictions in the predefined AD were deemed to be comparatively reliable, while predictions outside or near the boundary were interpreted with more caution. The AD assessment was, therefore, used as a predictor of reliability, and not as a biological activity assessment during compound prioritization.

#### 2.1.3. Feature Importance of SHAP-Based Model Applicability Analysis

**Figure 3:**
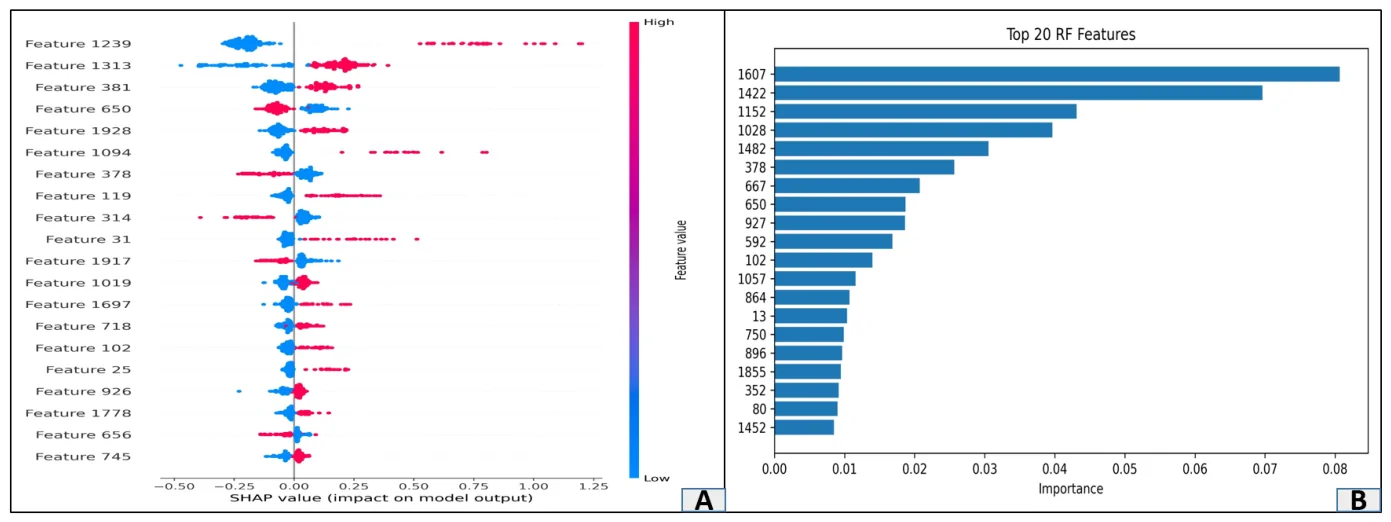
Feature importance analysis of a machine learning model. (**A**) represents the SHAP summary plot of top molecular fingerprint features ranked by how they affect the model output; each point represents a compound, and the color of the point represents the feature value (high in pink, low in blue), while the position of the point on the x-axis represents the SHAP value. (**B**) represents the Top 20 most important features identified by the random forest (RF) model in decreasing order of importance score.

To improve the interpretability of the machine learning predictions, SHAP (SHapley Additive exPlanations) analysis was used to identify molecular fingerprint features that contributed most strongly to the model predictions [13]. The SHAP values provide a model-specific measure of the contribution of individual fingerprint features toward the predicted activity and therefore help identify structural patterns associated with the model’s decision-making process [14]. SHAP analysis identified features 1239, 1313, 381, and 650 as the most influential fingerprint bits for the SHAP analysis (Figure 3A), with the presence of these fingerprint bits being associated with increased predicted potency (positive SHAP value) in general, while the presence of the 378 and 314 fingerprint bits was associated with decreased predicted pIC50 (negative SHAP value) [15]. The top features identified by random forest feature importance (Figure 3B) were partially overlapping but different (1607, 1422, 1152, 1028, and 1482), showing that there are some differences between impurity-based and SHAP-based importance metrics. Both methods agreed that 378 and 650 were consistently important features, which are likely to represent non-redundant, strong structural determinants of potency. It is recommended that these fingerprint bits be mapped back to the substructures they represent, using RDKit analysis, in order to get chemically interpretable SAR insights.

#### 2.1.4. Machine Learning Model Performance

**Figure 4:**
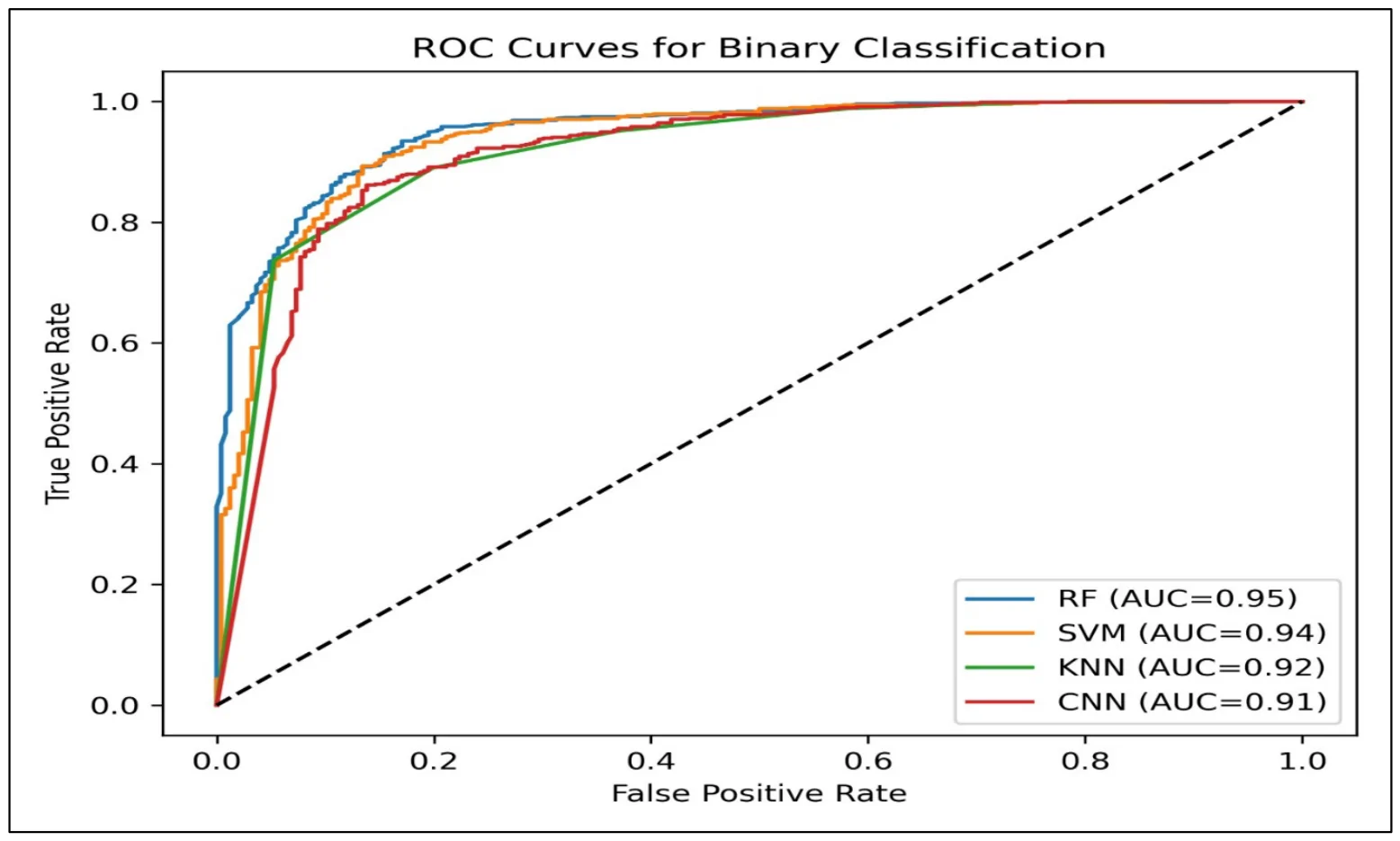
Receiver operating characteristic (ROC) curves for the effectiveness of various machine learning classifiers, namely random forest (RF), support vector machine (SVM), K-Nearest Neighbors (KNN), and Convolutional Neural Network (CNN) in binary classification of active and inactive compounds. AUC values represent the discriminating capability of each model, and the RF model has the highest discriminating capability (AUC = 0.95).

The performance of the machine learning models evaluated for the classification of the compounds into defined activity classes differed. Comparative assessment of the models allowed the selection of the most adequate model for further compound prioritization. The chosen model was then used to screen the chemical space for the search of compounds with a suitable activity profile, followed by molecular docking and further computational analysis. The biological activity indicated by the ML predictions was not used as an independent indicator of a biological response. Instead, they were used as a preliminary prioritization step in the integrated workflow, whereby the potentially promising compounds were further prioritized using structure-based and molecular simulation methods. This sequential approach minimized the chemical space and combined the ligand-based prediction with docking, ADME properties analysis, and molecular dynamics simulations. When interpreting the predictive performance of the models, the limitations of the dataset used and the lack of extensive external or scaffold-aware validation should be taken into account. The performance of the classification algorithms (RF, SVM, KNN, and CNN) was assessed in binary classification by ROC-AUC analysis (Figure 4). Random forest outperformed the other classifiers, with an AUC of 0.95, followed closely by SVM with an AUC of 0.94, and KNN and CNN had slightly lower but still impressive performance with AUCs of 0.92 and 0.91, respectively. The results from all models were well above the chance level, thereby demonstrating that meaningful signals are present in molecular fingerprint features to discriminate active from inactive compounds. This is in line with earlier published literature on QSAR, which favors RF and SVM over CNN for fingerprint representations with high dimensionality and sparse data, and for moderate-sized datasets.

### 2.2. Molecular Docking and Binding Affinity Analysis

**Figure 5:**
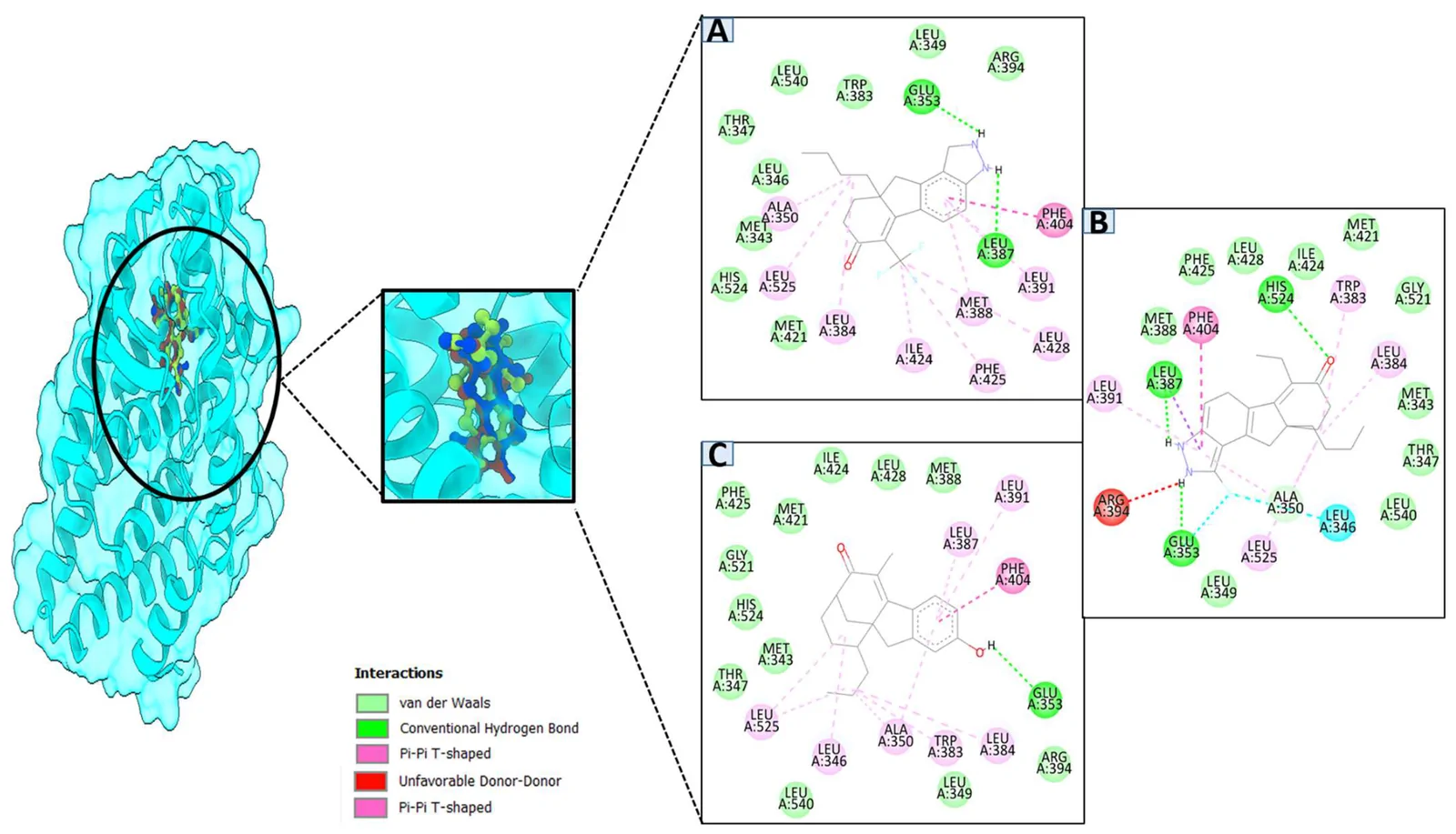
Docked binding poses of (**A**) Hit-1, (**B**) Hit-2, and (**C**) Hit-3 in the active site of ERα. Key hydrogen bonds (green dashed lines), hydrophobic interactions, and other contacts made to active site residues are shown in 3D and 2D interaction diagrams.

The molecular docking analysis was performed to assess the binding affinity of the ligands and ERα. The docking grid was centered on the experimentally characterized binding site of the target protein. The grid box dimensions were set to 20 × 20 × 20 Å, while the grid-center coordinates were defined according to the coordinates of the binding-site residues [16]. The Vina exhaustiveness parameter was set to 32 to provide more extensive conformational sampling. The receptor and ligands were prepared by adding hydrogens and assigning appropriate protonation states corresponding to approximately pH 7.4. The predominant biologically relevant tautomeric state was retained for each ligand. The stereochemistry of the input compounds was preserved throughout ligand preparation, and no stereochemical inversion was permitted during docking [17]. The analysis yielded three hits based on their binding affinity, as shown in Table 1. Based on the molecular docking results, all three hit molecules have docking sites within the canonical ligand-binding pocket of ERα and interact with the conserved residue Glu353 through a conventional hydrogen bond, consistent with the typical hydrogen-bonding network used by known ERα ligands, such as endogenous estrogen (Figure 5). Hit-1 (A) formed additional hydrogen bonds with Leu387 and a Pi-Pi T-shaped contact with Phe404, complemented by a large hydrophobic contact network. In the Hit-2 (B) pose, both Glu353 and His524 were engaged, mimicking the entire canonical estrogen-binding hydrogen-bonded triad, and a donor–donor interaction with Arg394 was also observed, an unfavorable interaction that could explain the conformational instability of this complex in the MD simulation. For Hit-3 (C), a single hydrogen bond was formed with Glu353, accompanied by Pi-Pi aromatic stacking and van der Waals contacts, with no unfavorable contacts, and it was found to be very stable according to all the MD-derived metrics. The results of these docking-level interactions provide a structural rationale that aligns with the dynamic stability ranking determined from the MD trajectory analyses, further supporting Hit-3 as the most promising lead candidate.

The reference control ligand used was 17β-estradiol, the physiological ligand for estrogen receptor alpha (ERα), and a biologically relevant standard to assess the biological activity of the identified ligands. The control was used as a physiological/reference ligand and not as an antagonist benchmark. The crystal structure of ERα used in this work (PDB ID: 6CBZ) is the crystal structure of the ERα in complex with estradiol (PDB ID: 6CBZ), which was determined experimentally at 1.65 Å resolution in the crystal structure. Thus, the observed binding mode for estradiol in the experiments was chosen as a structural model for the evaluation of the binding mode, key residue interactions, and binding stability for the investigated compounds [18]. The molecular docking of the control and ERα is shown in Figure S1. The RMSD of the control and the target protein was reported as 1.37 Å. Docking analysis showed favorable binding of the prioritized compounds within the ERα Y537S binding pocket. However, these findings indicate compatibility with the Y537S structural context and should not be interpreted as evidence of mutant-specific selectivity.

### 2.3. ADME and Drug-Likeness Prediction of Lead Compounds

ADME and drug-likeness properties of the three lead hits and the reference control were calculated in silico using the SwissADME platform (Table S1). All candidates met Lipinski’s Rule of Five with no violations and did not present any PAINS or Brenk structural alerts, indicating good drug-likeness and low assay interference and reactive toxicophores. All of the compounds were predicted to be well absorbed in the gastrointestinal tract and have the same bioavailability score of 0.55. Hit-1 (MW = 352.39, consensus LogP = 4.02) was selected for molecular weight-related “Lead likeness” violations and predicted “P-glycoprotein substrate liability”, which could potentially reduce sustained tissue exposure. Hit-2 (MW = 328.42) had the highest polar surface area (48.65 Å^2^) and was predicted to be P-gp negative, with decreased efflux liability. The most rigid and lipophilic Hit-3 (MW = 296.40 and consensus LogP = 4.82) was predicted to be permeable to the blood–brain barrier, which could be beneficial in reducing off-target exposure to the central nervous system in the context of ERα-targeted breast cancer treatment. The control compound, which represented the typical steroidal estrogen scaffold, had the lowest number of interactions in the whole structure and the highest synthetic accessibility, as would be expected for the unmodified reference compound. These results together suggest that all three hits possess good oral drug-likeness and scaffold modifications that make the predicted PK and permeability properties distinct from those of the endogenous hormone. The ADME analysis was primarily used to assess the pharmacokinetic and drug-likeness profiles of the candidate compounds. Specific toxicity endpoints, including hERG channel inhibition and hepatotoxicity, were not independently predicted in the present study and therefore warrant further evaluation using dedicated in silico toxicity models and experimental assays.

### 2.4. Molecular Dynamics (MD) Simulation Analysis

#### 2.4.1. RMSD and RMSF

**Figure 6:**
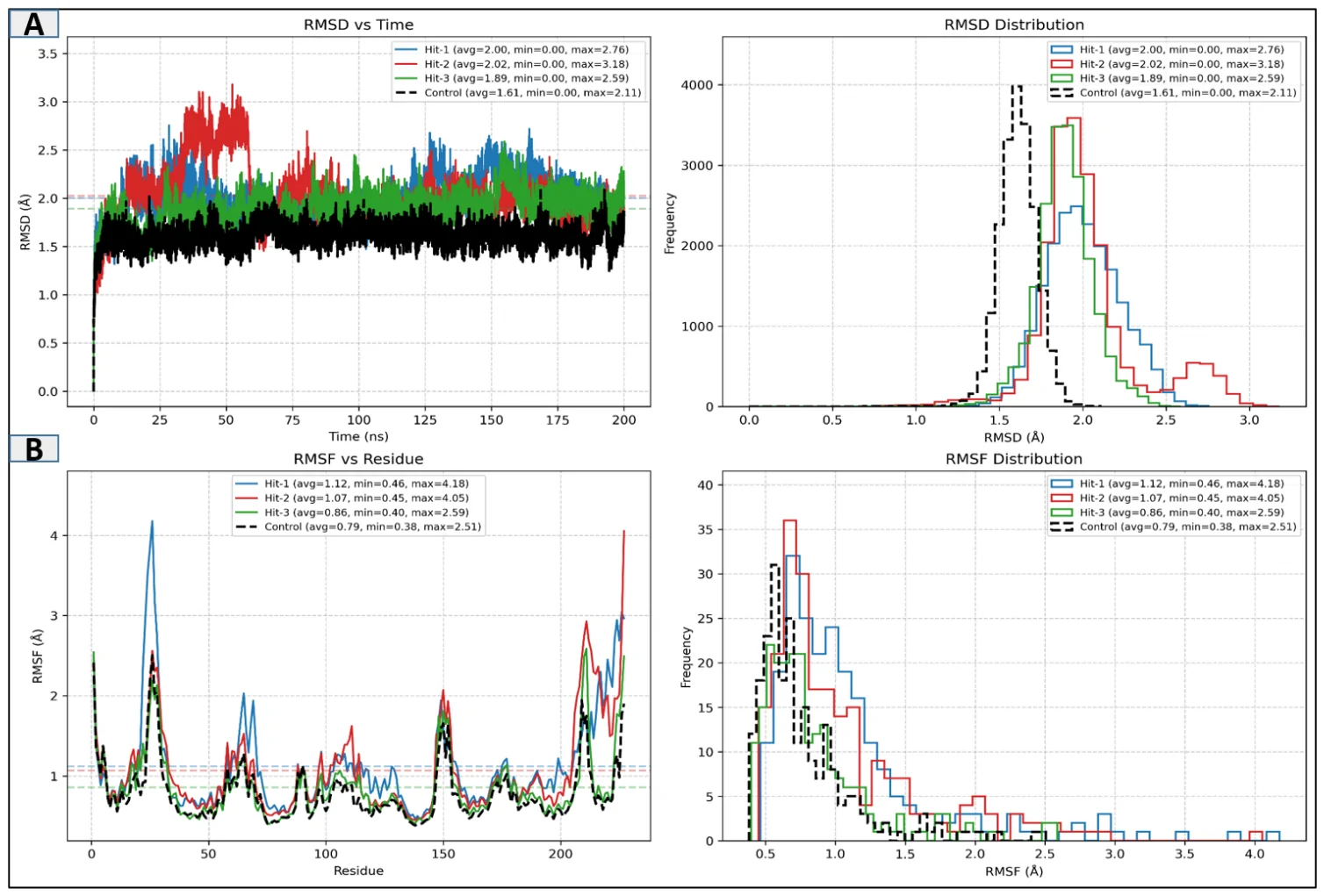
The RMSD of the protein backbone Cα atoms for the complexes of ERα with Hit-1, Hit-2, Hit-3, and control, computed over 200 ns of molecular dynamics simulation, shows that the protein structure is stable when bound to each compound (**A**). Plot of RMSF indicates the flexibility and stability of the binding pocket for each of the complexes over the course of the simulation (**B**).

To evaluate the stability of protein–ligand complexes, MD simulations (200 ns) were carried out for the three top hits versus a control [19]. The deviations of the control complex in this RMSD analysis (Figure 6A) were quite low, resulting in low variance throughout the simulation (control avg = 1.61 Å), while all three hits displayed moderately higher but bounded deviations (avg = 1.89–2.02 Å), showing overall stability of binding. The RMSD deviation of Hit-2 was found to be transient, with values ranging from 30–55 ns, with the maximum excursion being 3.18 Å, which implies that the protein underwent a conformational change before re-stabilization. Flexibility analysis at the residue level (RMSF) indicated that the systems exhibited flexibility in native flexible regions of the structure, but also showed some local destabilization in and around residue 25 (Hit-1) and 210–230 (Hit-2), C-terminal regions Figure 6B. Hit-3 was the most stable hit both globally and locally, compared to the control, and it could be considered the most conformationally stable hit amongst the three hits of this study.

#### 2.4.2. RoG and Beta Factor

**Figure 7:**
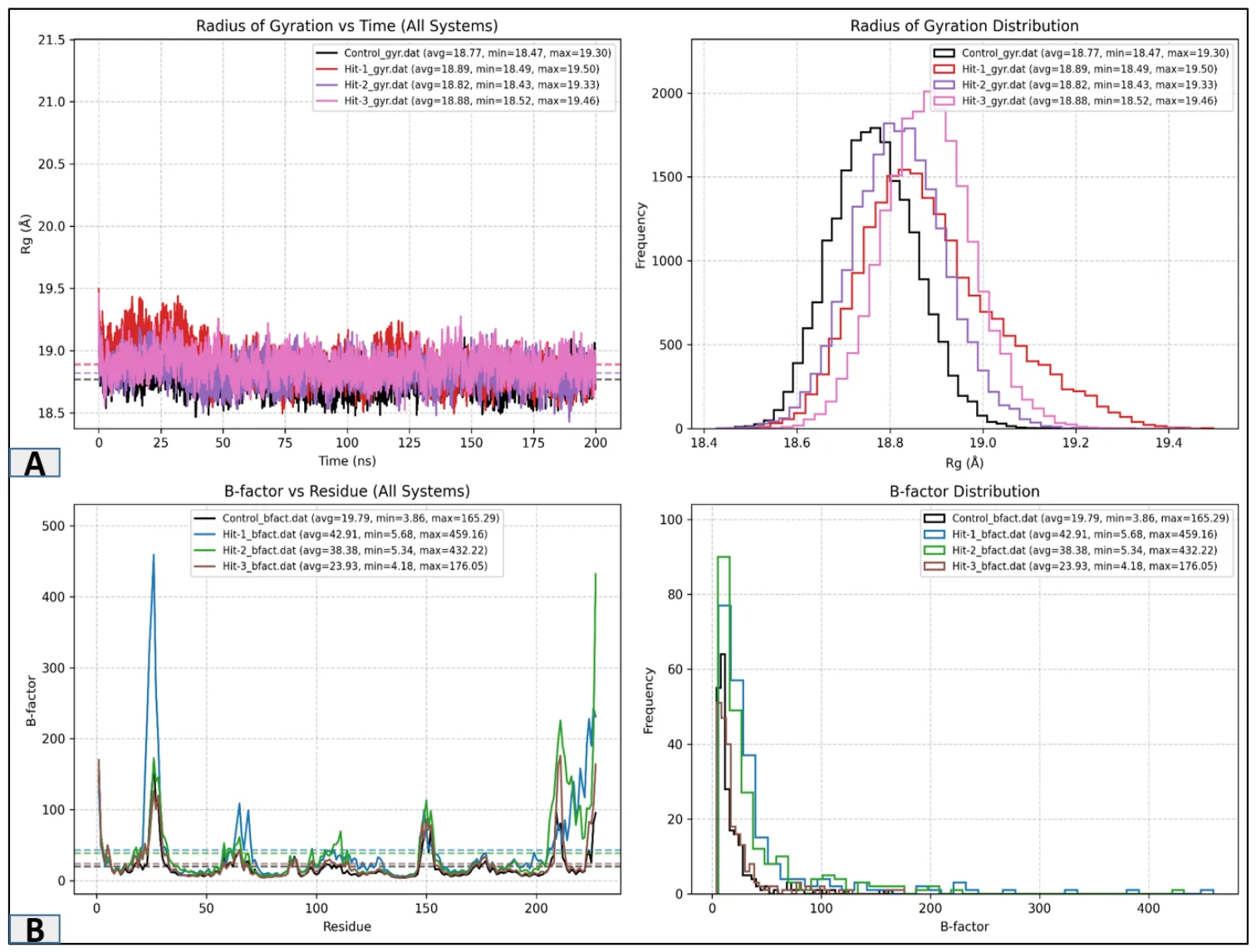
The radius of gyration (RoG) of the Hit-1, Hit-2, Hit-3, and control complexes is plotted against simulation time, indicating the compactness and structural integrity of the ERα during the 200 ns MD simulation (**A**). Per-residue B-factor profile of ERα in complex with Hit-1, Hit-2, Hit-3, and control, showing regions of flexibility and rigidity for the four systems (**B**).

No global unfolding events were observed during the 200 ns simulation, as all four complexes remained structurally compact with RoG values that were relatively constant throughout the simulation and had average values of 18.77 Å (control) to 18.89 Å (Hit-1) (Figure 7A). The residue-specific destabilization was confirmed in the B-factor analysis (Figure 7B), which was consistent with the RMSF results, with a greater increase in local flexibility in Hit-1 near residue 25 (max B-factor = 459.16) and in Hit-2 at the C-terminal region (residues 210–230, max B-factor = 432.22). When comparing both metrics (avg B-factor = 23.93 vs. 19.79), Hit-3 was the closest among all the evaluated hits to the control.

#### 2.4.3. Ligand RMSD and SASA

The RMSD at the protein level (Figure S2), which is a measure of binding pose stability independent of overall protein motion, indicated the maximum instability with Hit-2, showing a temporary deviation of ~3.0 Å from 25 to 55 ns, which could correspond to binding pose rearrangement, similar to the protein-level RMSD deviation seen for this system. Hit-3 was most similar to the binding mode of the control ligand (1.89 Å, compared to 1.61 Å), which was further evidence for a stabilized and well-maintained binding mode. The exposure to solvents was modestly higher for Hit-1 (avg = 13,026 Ų) and Hit-2 (avg = 13,053 Ų) than for control (avg = 12,860 Ų), as seen in the SASA analysis (Figure S2); this was also reflected in the localized flexibility increases shown in the RMSF/B-factor analyses, whereas the exposure for Hit-3 was closest to control (avg = 12,996 Ų). Combined, these results suggest that the Hit-2 binding pose would be a good target for further analysis, such as free energy decomposition or visual trajectory analysis, while the Hit-3 binding pose would be more conformationally and energetically stable.

#### 2.4.4. The Free Energy Landscape (FEL) and Principal Component Analysis (PCA)

**Figure 8:**
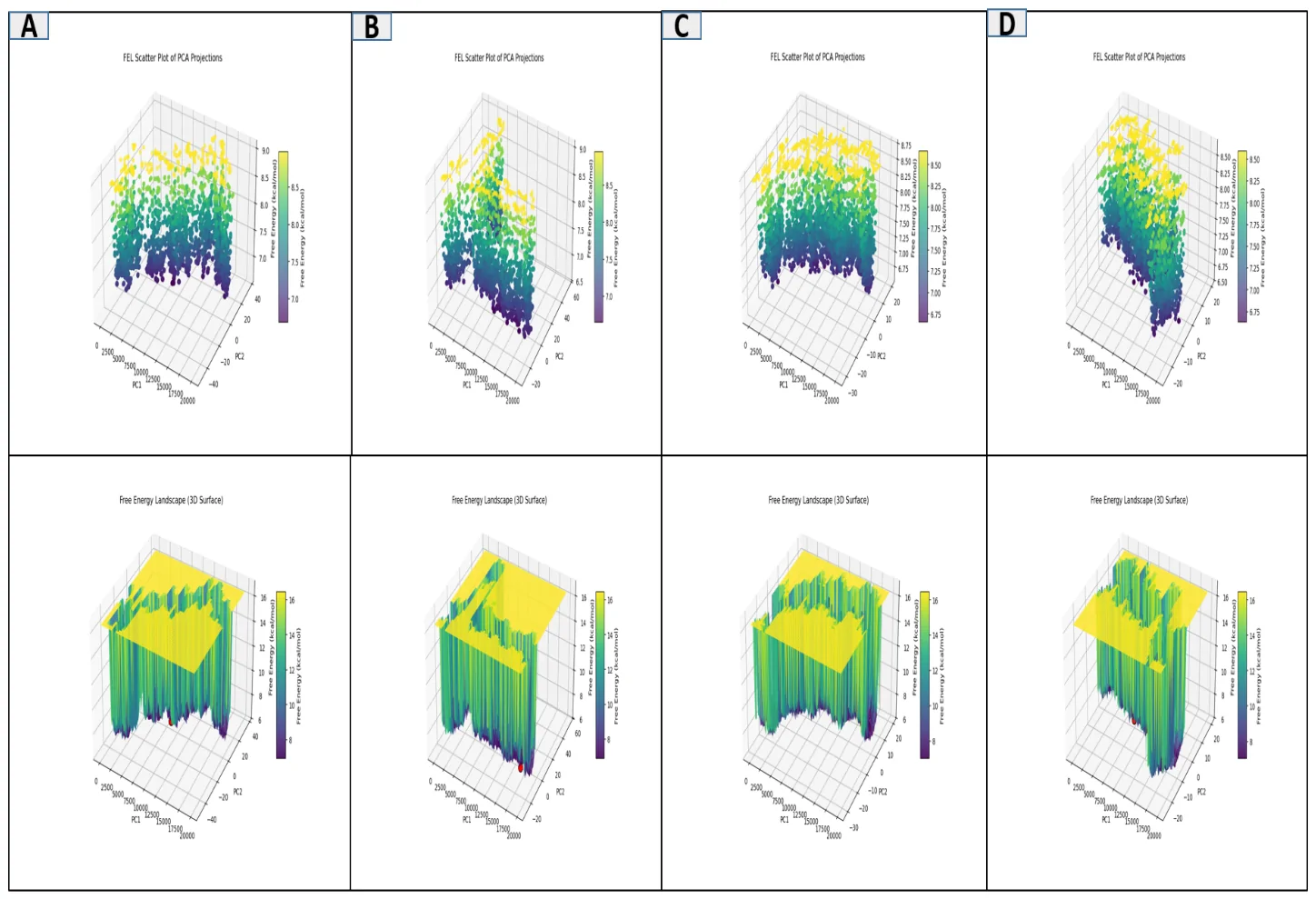
Principal component analysis (PCA) of the MD trajectories for Hit-1, Hit-2, Hit-3, and control with the projection of protein motion onto the first two principal components (PC1 and PC2). The points correspond to individual simulation frames and show the conformation space sampled for ERα in each of the complexes. The (**A**) Hit-1, (**B**) Hit-2, (**C**) Hit-3, and (**D**) control complexes are built from PC1 and PC2 as reaction coordinates to create the Gibbs free energy landscape (FEL). The color gradient shows the relative free energy (kcal/mol) for each basin of the conformational space of ERα; low-energy basins (thermodynamically stable states) are shown in dark blue/purple. The red dot represents energy minima.

To characterize the conformational dynamics of the individual protein–ligand complexes beyond simple RMSD/RMSF, FELs were generated by projecting the 200 ns MD trajectories onto the first two principal components (PC1, PC2) of the Cα backbone motion, and estimating the relative free energy by Boltzmann inversion of the conformational population density (Figure 8) [20]. All four complexes showed rugged multi-basin landscapes instead of single deep funnels, which indicates flexibility of the protein over the timescales simulated (Figure 8). The differences in the conformational sampling of the three hit complexes versus the control were seen from the PCA and free energy landscape (FEL) analyses. Hit-1 (A) had several low-energy regions that were distributed across the conformational space, which suggests that Hit-1 (A) sampled multiple favorable conformations. The distribution of low-energy conformations was more concentrated in Hit-2 (B), while Hit-3 (C) had a broader distribution of conformations with several low-energy regions. The conformational distribution in the control (D) was more restricted. On the whole, the FEL profiles suggest that the hit complexes were in energetically favorable conformational states throughout the simulations, but with multiple basins, a certain conformational heterogeneity can be assumed. Although these results are not direct evidence for differences in binding affinity or stability, they do complement existing evidence that there are differences in conformational dynamics between the studied complexes.

#### 2.4.5. DCCM

**Figure 9:**
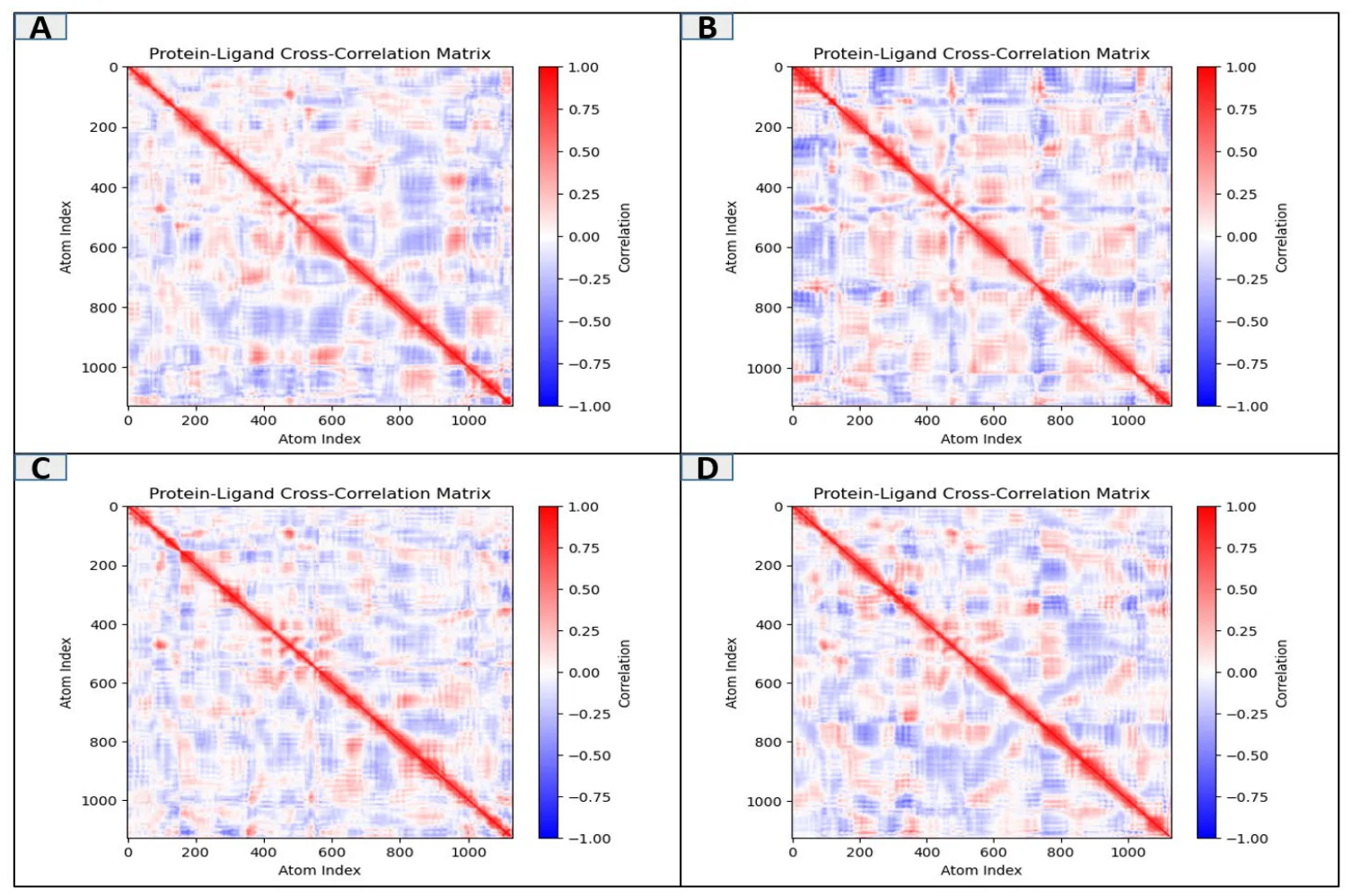
DCCM analysis of Cα atoms for the (**A**) Hit-1, (**B**) Hit-2, (**C**) Hit-3, and (**D**) control complexes derived from the 200 ns MD trajectories. The color scale represents the degree of correlated (red, positive values) and anti-correlated (blue, negative values) motions between residue pairs, with values closer to +1 indicating highly correlated (same-direction) motion and values closer to -1 indicating highly anti-correlated (opposite-direction) motion of ERα upon ligand binding.

To understand the correlated and anti-correlated atomic movements in each protein–ligand complex along the MD trajectory, the dynamic cross-correlation matrix (DCCM) analysis was performed [21]. The general domain architecture and the type of collective motions in all complexes were found to be broadly conserved, showing that the basic domain architecture and the collective motions remained unchanged, irrespective of the bound ligand. The heterogeneous patterns of correlated and anti-correlated motions were confirmed for all systems with the DCCM analysis. The motions of the various protein regions were found to be both positively correlated (red) and negatively correlated (blue) for Hit-1 (A), Hit-2 (B), and Hit-3 (C), suggesting that there were motions that were coordinated and oppositely coordinated between different protein regions during the simulations (Figure 9). The correlation pattern was similar in the control (D), and the distribution and intensity of correlated regions varied in the different systems. Taken as a whole, the DCCM results reveal a difference in the dynamic coupling of protein regions when the ligand is bound, and the patterns observed should be considered in terms of changes in correlated motions instead of an increase in binding affinity or stability.

#### 2.4.6. RDF

**Figure 10:**
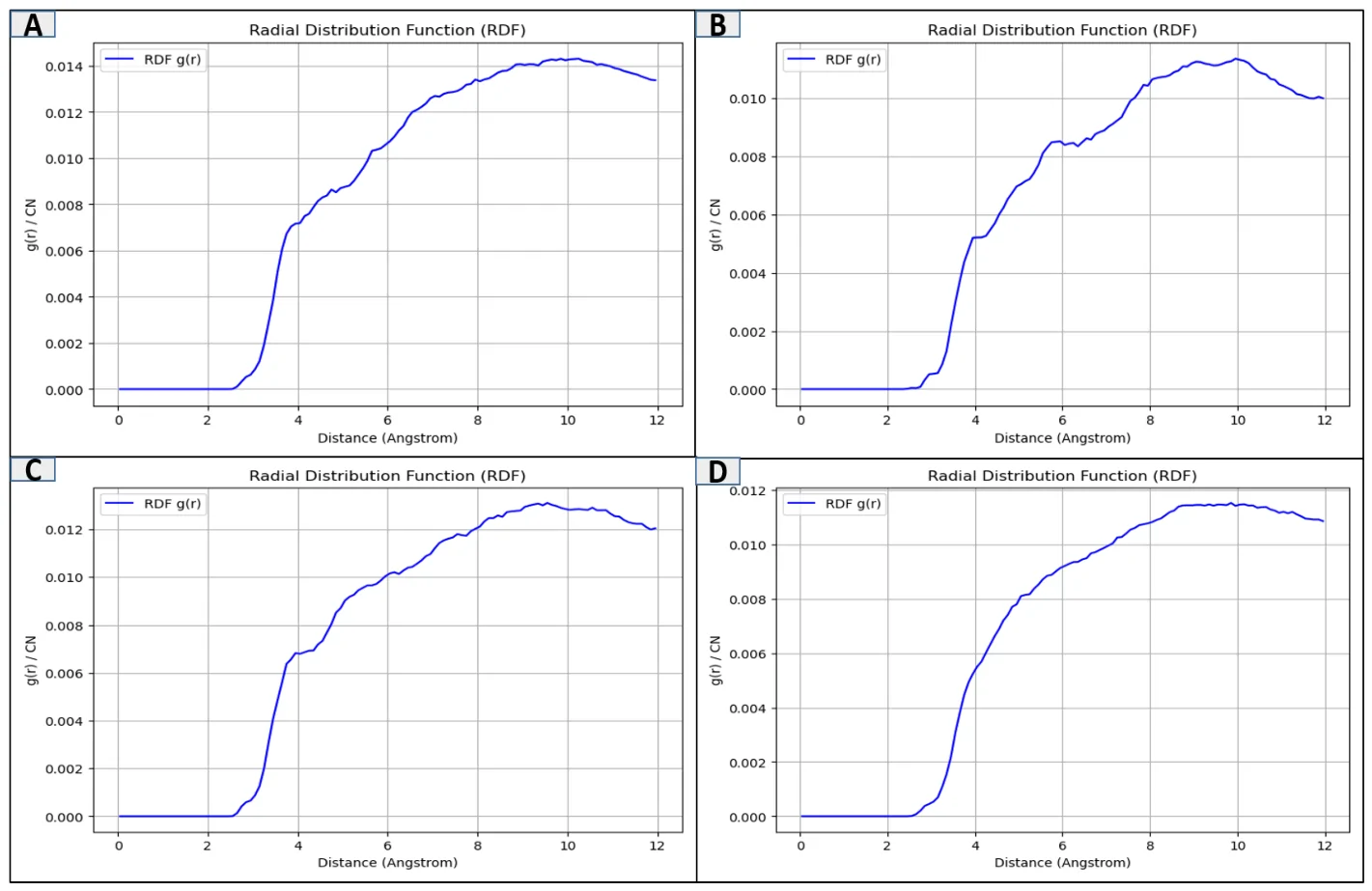
Radial distribution function (RDF), g(r), of ERα with Hit-1 (**A**), Hit-2 (**B**), Hit-3 (**C**), and control (**D**) for the probability of atoms/residues of the target protein being encountered at a particular distance r, highlighting the strength and persistence of important non-covalent interactions found in each complex.

The local packing density of the protein atoms around each ligand has been calculated using the radial distribution function (RDF) analysis [22] (Figure 10). All complexes showed a very similar profile; at densities below ~2.5 Å, the density was negligible, which reflects the volume of the excluded ligand; the density increased sharply between 3 and 8 Å, as first-shell protein atoms are encountered; and a broad density maximum occurred between 9 and 10 Å. Hit-2 had the highest packing density (g(r)/CN ≈ 0.0152), but more unstable local contacts in the range of 5–7 Å, as seen in the RMSD analysis of the transient contacts of the ligand, indicating close, but not always consistent, contacts. The peak density for control (D) was comparatively flatter and plateau-like, which may reflect two nearly coincident coordination shells, while that for Hit-3 (C) was the lowest and may indicate the local packing environment to be somewhat looser. This packing difference is generally in agreement with the relative stability results obtained from RMSD, RMSF, and free energy landscape analysis. The spatial distribution of selected ligand–residue atom pairs throughout the MD trajectories was analyzed using the RDF. RDF features were not considered to be direct measures of binding affinity but rather as being indicative of the preferred spatial proximity of the atom pairs analyzed.

#### 2.4.7. Salt Bridges Analysis

To monitor the stability and consistency of the electrostatic interaction network for each complex, the number of salt bridges at each frame was monitored throughout the 200 ns trajectory [23] (Figure S3). In Hit-2 (B), the electrostatic network remained stable, and the overall distribution was mostly unimodal with a peak around 15–17 salt bridges, which suggests a stable and well-maintained electrostatic network throughout the simulation. Conversely, Hit-1 and Hit-3 (A, C) revealed bimodal distributions, indicating the occurrence of regular changes between two semi-stable salt-bridge configurations. The multi-modal behavior of control (D) was most apparent, where there was a clear and frequently populated high-salt-bridge state (22–24 bridges) as well as the prominent baseline state (13–14 bridges), suggesting frequent switching to a more widely coordinated electrostatic configuration. This is consistent with the transient conformational instability seen in this system in the ligand RMSD and free energy landscape analyses, implying that the conformational shifts observed could be indicative of a short period of time spent in an alternate binding or contact mode. The observed interaction populations do not give structural information about the persistence of these interactions in the sampled trajectories, but provide secondary information about these interactions.

#### 2.4.8. MMPBSA/GBSA

The selected simulation trajectories were used for MD simulation, and the estimated binding free energies were subsequently calculated using the MMPB/GBSA analysis [24]. The comparative estimates of the binding energetics of the investigated protein–ligand complexes were obtained by the calculations of MMPB/GBSA (Table 2). Among the compounds analyzed, Hit-1 exhibited the best estimated binding energy. These values must be viewed as relative computational estimates, and not as binding affinities or thermodynamic free energies, in the context of the applied MMPB/GBSA framework. In particular, the present calculations do not explicitly include conformational entropy, and uncertainty estimates were not calculated. The results were therefore interpreted to aid computational prioritization of ligands for comparative purposes only and not as a measure of absolute ligand binding strength or thermodynamic stability. Hit-1 had the best binding energy (net MMPB/GBSA −129.59/−128.95 kcal/mol) among all novel hits investigated and thus forms the most stable complex among the others. The binding of Hit-2 and Hit-3 was also favorable, with MMPBSA net energies of −115.86 kcal mol^−1^ and −114.39 kcal mol^−1^, respectively. The higher affinity value of the compounds obtained in the novel group was observed, as the net energy was −100.99 kcal mol^−1^ for the control compound, suggesting that the novel hit compounds are more stable in the binding site of the target. Based on these results, the compounds have good binding activity with respect to the target compared to the reference control.

## 3. Discussion

The current study provides a structure-based computational and machine learning investigation toward the discovery of novel computationally prioritized hits for the estrogen receptor α (ERα) Y537S mutant, which is clinically important for endocrine-resistant breast cancer [25]. Using machine learning to guide virtual screening, a list of three potential compounds, Hit-1, Hit-2, and Hit-3, and a reference control molecule were selected to be studied in detail to investigate their binding behavior inside the ligand-binding domain (LBD) of the mutant receptor. All three hit compounds were found to be effective in binding the Y537S LBD pocket using molecular docking followed by MD simulations, with the compounds binding in a way that occupied the pocket and thus disrupted the constitutively active Y537S helix-12 conformation. Hit-1 exhibited the most favorable binding profile of all the hits, with multiple short-lived hydrogen bonds and hydrophobic interactions with residues important to the receptor conformation. The binding was also found to be stable, albeit with somewhat less favorable binding free energies in Hit-2 and Hit-3. The control compound, in contrast, exhibited less persistent interactions and more conformational drift in the binding pocket, which is consistent with its ability to potentially counteract the shift toward the agonist conformation driven by the Y537S mutation to a lesser extent. The dynamic stability of the ligand-receptor complexes was confirmed by MD trajectory analyses. Both RMSD and RMSF profiles showed that the overall shape and structure of the LBD remained stable during simulations, showing significantly less flexibility in the loop and helix regions around the binding pocket, especially in the Hit-1 complex. The more detailed analysis of the contacts between the ligands and the polar residues indicated by RDF was consistent with the persistence of these contacts, with Hit-1 and Hit-2 exhibiting sharp, well-defined peaks suggesting long-lived hydrogen bonding. The large-scale conformational motions of the receptor were inhibited by Hit-1 binding and expanded along the principal modes in the PCA analysis of the control. These were confirmed by the free energy landscape, where the most tightly bound complexes were observed, and Hit-1 was in a deep, well-defined conformational energy minimum, corresponding to a computationally prioritized compound.

The binding free energy calculations with MMPBSA/GBSA further confirmed these observations, as all three hit compounds had more favorable binding free energies than the control. The energy terms decomposed showed that both electrostatic and van der Waals interactions were significant, suggesting that both charged and non-polar interactions of the residues play a role in producing high-affinity binding with mutant specificity. Together, these findings indicate that the hits identified, especially Hit-2 and Hit-3, are good candidates for targeting the Y537S LBD for blocking endocrine resistance. Hydrophobic packing, electrostatic complementarity, and hydrogen bonding seem to be central to their inhibitory capabilities, and retention of the receptor’s overall fold means that they interact by targeted conformational modulation, rather than nonspecifically destabilizing the receptors.

A 2025 review of AI in structure-based drug discovery mentioned the use of ML algorithms to improve scoring functions for more accurate binding-affinity prediction than the traditional empirical/force-field approaches, and that a typical workflow would involve docking a large library of compounds to a target, and then running MD simulations to check if the binding pocket was stable, followed by the calculation of the MMPBSAs of these docked poses, which would be used to rank the poses, and then selecting the top-scored poses to be tested experimentally [26]. A similar study from 2024, using MD simulation against the Y537S + F404V Double-Mutant Estrogen Receptor Alpha, also synchronizes with the recent work [27]. Other relevant studies were conducted by the authors of [28]. Furthermore, a study from the Cancer Discovery team in 2024 revealed that individuals with a baseline ESR1 F404 mutation had a shorter median progression-free survival (PFS) on fulvestrant than those without the mutation, highlighting the clinical importance of targeting this particular mutant [29].

The rapid prioritization of bioactive compounds from large chemical libraries has become possible with recent developments in artificial intelligence (AI) and machine learning (ML), creating a new paradigm for computer-aided drug discovery (CADD). Ligand-based ML and deep-learning methods, for example, can use molecular descriptors, fingerprints, and experimentally obtained bioactivity data to find candidates that are likely to be good before using more computationally demanding structure-based methods. These are complemented with structure-based virtual screening, which assesses binding modes and interactions in the target binding site for the prioritized compounds, and then additional information can be gained from molecular dynamics (MD) simulations of the protein–ligand complexes [30]. The workflow that was used in the present work, which proves useful in narrowing down the vast chemical space and has the advantage of combining the use of ML for prioritizing the compounds followed by molecular docking, ADME/drug-likeness evaluation, MD simulation, and the obtainment of the end-point binding free energy, constitutes a step-by-step approach. The same kind of integrated and AI-assisted virtual screening has been shown to be capable of making effective predictions and to be a powerful way of combining data-driven prediction with physics-based structural assessment, to enhance hit identification and to reduce the computational workload of screening large chemical libraries [31].

The favorable interactions observed with ERα Y537S support the potential of the prioritized compounds as candidate binders. However, mutant specificity cannot be established without a parallel comparison with wild-type ERα. Comparative computational and experimental studies of wild-type and Y537S ERα are therefore required to determine mutant selectivity and confirm biological activity.

This study combines hit identification using machine learning approaches with structure-based validation to provide a complete computational strategy from predictive screening to mechanistic (energetics) confirmation of binding behavior, which serves as a solid basis for selection of candidates for further experimental testing on the downstream side. One of the limitations of the current MD analysis is that only a single 200 ns trajectory was analyzed for each protein–ligand system. The trajectories were helpful in providing comparative information about the structural behavior of the complexes, but it was not possible to establish convergence or sample the conformational space of these systems exhaustively from a single trajectory. Thus, the differences in RMSD, RMSF, hydrogen-bonding, interaction persistence, and FEL profile values observed should be considered as comparative observations, not statistically significant differences between the compounds, as it concerns the trajectory. Additional evidence for the reproducibility and robustness of the observations would come from independent replicate simulations and longer or higher-quality simulations.

## 4. Material and Methods

**Figure 11:**
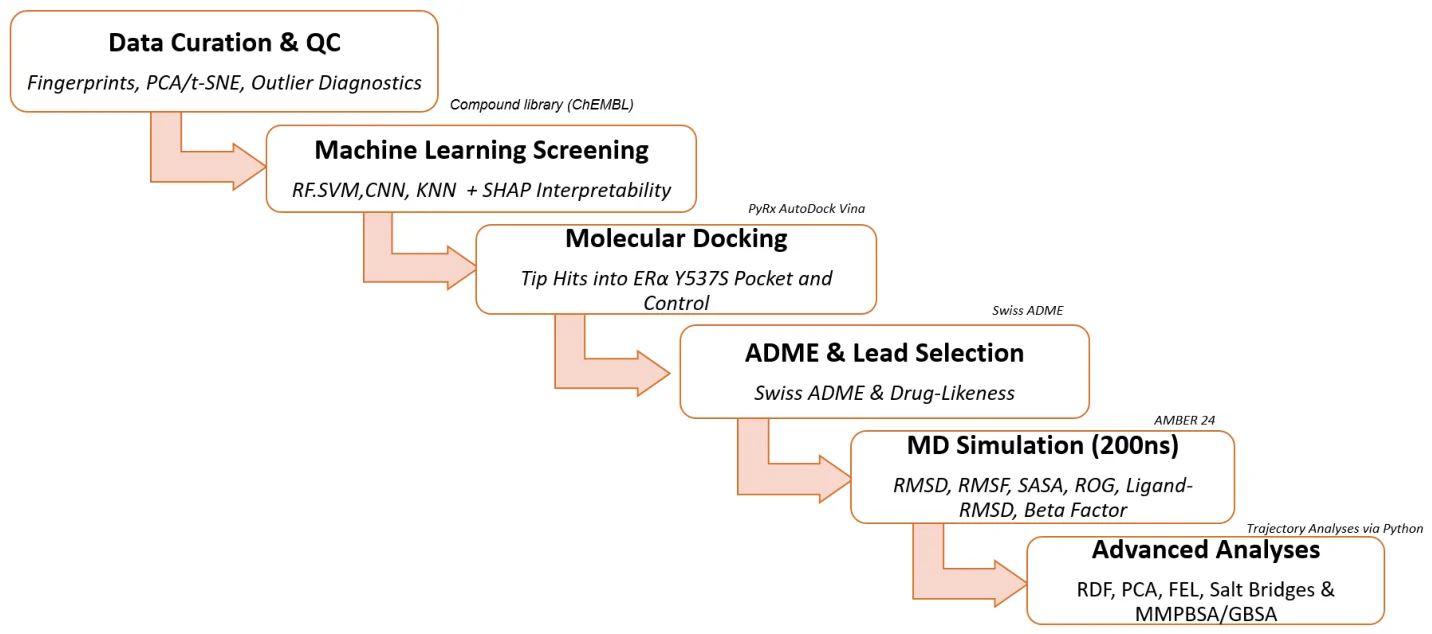
Workflow of the computational pipeline for identifying ERα Y537S computationally prioritized hits. Compounds curated from ChEMBL underwent fingerprint-based chemical space analysis and outlier QC, followed by machine learning classification (RF, SVM, CNN, and KNN) with SHAP-based interpretability. Top hits and a control were docked into the ERα Y537S pocket (PyRx/AutoDock Vina v0.8), filtered by SwissADME drug-likeness, and subjected to 200 ns MD simulations (AMBER 24) evaluating RMSD, RMSF, SASA, ROG, ligand RMSD, and B-factor. Advanced trajectory analyses (RDF, PCA, FEL, salt bridges, and MM-PBSA/GBSA) further characterized binding stability and thermodynamics.

In this study, an integrative computational pipeline was used, which comprised molecular docking, quantitative structure–activity relationship (QSAR)-guided virtual screening, and long-timescale molecular dynamics (MD) simulation to identify and mechanistically characterize potential lead compounds against the target protein(s) of interest. The whole process, consisting of successive and iterative analytical steps, is depicted in Figure 11.

### 4.1. Artificial Intelligence (AI) for Screening of the Compounds

Bioactive compounds against the target protein were extracted from the public chemical and bioactivity repository (such as ChEMBL) to create a structure–activity relationship set. The dataset was cleaned and curated by eliminating duplicate entries as well as inorganic compounds and mixtures, and then by standardizing molecular structures using RDKit. Compounds with missing or invalid activity or structure were discarded. Measurements depicting the same pharmacological parameter and biological endpoint were considered for individual predictive models, and IC50, Ki, or EC50 were not treated as interchangeable observations for model training [32]. Molecular descriptors and fingerprints were calculated by PaDEL-Descriptor v2.21 and RDKit software 2026.03.6, and selected based on protein expression profiling results in order to highlight the most relevant features for the target pathway [33]. To ensure the quality and consistency of the dataset, the compounds were subjected to a systematic curation procedure. Duplicate compounds and entries with missing or ambiguous activity information were removed. The curated dataset was then randomly split into a training set (80%) and a test set (20%), and various regression or classification models, such as multiple linear regression, random forest, and support vector machine, were used to construct the QSAR models [34]. To validate the model robustness and predictive reliability, internal cross-validation [Q^2^ > 0.5] and external validation [R^2^pred > 0.6] were performed, according to OECD principles of model validation for QSAR [35]. Applicability domain along with outlier assessment was conducted via Williams plots, Cook’s distance, and Mahalanobis distance plots. Out of the 35,000 compounds, 3353 compounds were found active and were further processed for molecular docking studies.

### 4.2. Molecular Docking

The three-dimensional structure of the target protein with the specific mutation was downloaded from the RCSB Protein Data Bank (PDB ID): 6CBZ [36]. The retrieved structure was processed for the removal of crystallographic water molecules and co-crystallized ligands, the addition of polar hydrogen atoms, and the application of Kollman partial charges with the help of UCSF Chimera [37]. The energy minimization of ligand structures selected from the QSAR-curated dataset was performed using the MMFF94 force field in Open Babel v3-2-1 and then converted to PDBQT format for docking. The molecular docking was performed using AutoDock Vina, which is an integrated component of PyRx 0.8 [38], with a grid box defined to cover the predicted or experimentally defined binding site, and docking exhaustiveness was set to 50 per molecule [16]. The grid dimensions were set as such to cover protein active regions, which include helices (H3, H5, H8, H11, and H12). The grid box was set around active site residues like Leu346, Thr347, Ala350, Leu387, Met388, Leu391, Arg394, Phe404, Glu353, Leu525, and Tyr537/Ser537 [18]. Docked poses were ranked based on their predicted binding energy, key hydrogen bonds, and hydrophobic interactions with active-site residues and adherence to Lipinski’s Rule of Five. The docking protocol utilized here was validated by removing co-crystallized ligands from PDB ID: 4QDI and redocked binding to the MurF protein. The redocked UDP and ATP molecules to MurF showed almost the same binding conformation as that of the crystal structure, with RMSD values of 0.12 Å and 0.14 Å, respectively [39]. The predicted ADME properties as calculated by SwissADME were used to shortlist the best possible compounds [40]. Those lead compounds, which were selected from the top in this process, were forwarded to an MD simulation.

### 4.3. ADME for Shortlisting Three Lead Compounds

The pharmacokinetic and drug-likeness properties of the selected lead compounds were predicted using the SwissADME web server (http://www.swissadme.ch) accessed on 4 April 2026 from the Swiss Institute of Bioinformatics [41]. The molecular docking results generated the canonical SMILES of each of the top lead compounds that were submitted to the server for evaluation of absorption, distribution, metabolism, and excretion (ADME) parameters [42]. The physicochemical properties, such as molecular weight, number of hydrogen bond donors and acceptors, topological polar surface area (TPSA), and lipophilicity (consensus Log Po/w), were calculated for each candidate to estimate the oral bioavailability and drug-likeness of these compounds according to Lipinski’s Rule of Five. The BOILED-Egg model, which classifies compounds by their position in lipophilicity–polarity space, was used to predict gastrointestinal (GI) absorption and blood–brain barrier (BBB) permeability, and the potential for passive absorption was assessed visually and quantitatively [43]. The BOILED-Egg represents a general physicochemical prediction parameter to demonstrate compounds’ gastrointestinal absorption and BBB permeability. The test is not ERα-specific and aids in preliminary ADME prioritization of compounds rather than evidence of efficacy or safety [44]. The compounds were also tested for their ability to inhibit the cytochrome P450 isoenzymes (CYP1A2, CYP2C19, CYP2C9, CYP2D6, and CYP3A4) and to act as substrates for P-glycoprotein (P-gp), an efflux transporter that can affect the absorption and distribution of drugs. Overall drug-likeness was also estimated using the calculated Log Kp value, and further assessment was made using multiple criteria predictive filters such as Ghose, Veber, Egan, Muegge, and a bioavailability score, which reflects the probability of achieving at least 10% oral bioavailability in a rat model. The compounds with favorable ADME properties, with a low number of violations of the drug-likeness filters, were selected as potential candidates to be further investigated in terms of molecular dynamics (MD) simulation and binding free energy analysis.

### 4.4. MD Simulation

The selected top Hit–ERα complexes were subjected to all-atom molecular dynamics (MD) simulations with AMBER software using the ff14SB force field for protein and AM1-BCC partial charges for the ligand using the General AMBER Force Field (GAFF2) [45]. The complexes were each placed in a TIP3P explicit water box, with a minimum buffer distance of 10–12 Å between the protein surface and the edge of the box, and counterions (Na^+^ and Cl^−^) were added to the system to approximate the physiological ionic strength [46]. Before the systems were simulated, energy minimization was performed for each system, followed by slow heating from 0 to 300 K at constant volume (NVT ensemble) and then equilibration at constant pressure (NPT ensemble) at 1 atm [47]. Production runs were then performed, with a total simulation time of 200 ns, under NPT conditions at 300 K and 1 atm, with a 2 fs integration time step, with SHAKE applied for constraining bonds involving hydrogen atoms [48]. Trajectory frames were saved every time step during the production run and then post-simulated with Python scripts v3.

### 4.5. Trajectory Analysis

#### 4.5.1. RMSD, RMSF, Beta Factor, RoG, Ligand RMSD, and SASA Analysis

Each simulated complex was evaluated for structural stability and flexibility by calculating the root mean square deviation (RMSD) of backbone atoms with respect to the minimized complex; the root mean square fluctuations (RMSF) of individual residues were used to identify the flexible and rigid structural regions; the radius of gyration (RoG) was calculated to monitor the overall compactness of the complex, and the solvent-accessible surface area (SASA) was calculated to monitor changes in surface exposure along the simulation trajectory. Residue-level thermal mobility was assessed using beta-factor analysis, whose trends were cross-validated with the corresponding RMSF profiles, while positional stability of the ligand within the binding pocket was independently monitored by calculating the ligand RMSD along the 200 ns trajectory [49].

#### 4.5.2. PCA and FEL

The collective motions (CMs) of the backbone Cα atoms were then analyzed via principal component analysis (PCA) using a Python script to identify the major modes of collective motions, and the free energy landscape (FEL) was calculated using the first two principal components, from which the most energetically favorable conformational states of the complex were identified [50].

#### 4.5.3. DCCM and Salt Bridges

In an attempt to understand whether there was allosteric communication within the complex, a dynamic cross-correlation matrix (DCCM) was generated, which not only captured positively correlated motions of the residues but also negatively correlated motions, and electrostatic stability of the complex was additionally explored by the analysis of salt bridge formation, using a distance cutoff of 4 Å between oppositely charged residue atoms [51].

#### 4.5.4. RDF

The spatial distribution of the various atoms of the solvent and the ligand around key active-site residues was characterized by calculating the radial distribution function (RDF) [52].

#### 4.5.5. MMPBSA/GBSA

Finally, the binding free energy for each of the top-ranked complexes was estimated using the Molecular Mechanics Poisson–Boltzmann/Generalized Born Surface Area (MMPBSA/MMGBSA) method with the aid of the MMPBSA.py module of AMBER, using snapshots from simulation trajectories taken at regular intervals of 0.02 ns (collected 10,000 frames) [24]. The solute dielectric, solvent dielectric, ionic strength, and SASA probe radius values were set to 1.0, 80.0, 0.150 M, and 1.4 Å, respectively. The entropy and enthalpy energy contributions were not considered due to their extensive computational power consumption. The obtained binding energy is an approximate comparative estimate, and so are the absolute experimental binding free energy values.

## 5. Conclusions

This study proposes an integrated machine learning and structure-based computational strategy to identify novel computationally prioritized hits of the ERα Y537S mutant associated with endocrine-resistant breast cancer, testing three compounds prioritized with machine learning against a reference compound. The computational results taken collectively provide complementary evidence for prioritizing the identified compounds over definitive evidence of experimental efficacy or binding affinity. The integrated workflow allowed the prediction of molecules with ML/QSAR, molecular docking, ADMET profiling, structural analysis based on MD simulation, and comparative MMPB/GBSA in order to identify molecules with favorable computational properties. Of the screened candidates, Hit-1 had the best comparative MMPB/GBSA score, and Hit-3 had comparatively good conformational behavior using a trajectory. Such results may be considered to be computational evidence for their consideration as candidates to be prioritized; however, they should not be interpreted as evidence of experimental binding affinity or thermodynamic stability, antagonist activity, or mutant specificity. To test the predicted interactions and the biological potential of these compounds, experimental biochemical and cellular studies will be required. The compounds identified by a machine learning and structural computational pipeline provide valuable computationally shortlisted leads for the rational design of next-generation therapeutics against endocrine-resistant breast cancer harboring ERα mutations. Based on the computational evidence provided here, these hits might be advanced toward experimental validation and/or optimization. Furthermore, the identified compounds showed favorable predicted binding within the ERα Y537S structural context. Nevertheless, mutant-specific selectivity cannot be concluded from the present analysis. Further wild-type-versus-Y537S comparison and experimental validation are required to confirm their selectivity and biological potential.

## Figures and Tables

**Table 1:** Contains the list of the three identified hits and the control, along with their chemical name, structures, and binding affinities. The control was used as a physiological/reference ligand and not as an antagonist benchmark.

Compound Rank	Compound Chemical Name	Chemical Structure	Binding Affinity
Hit-1	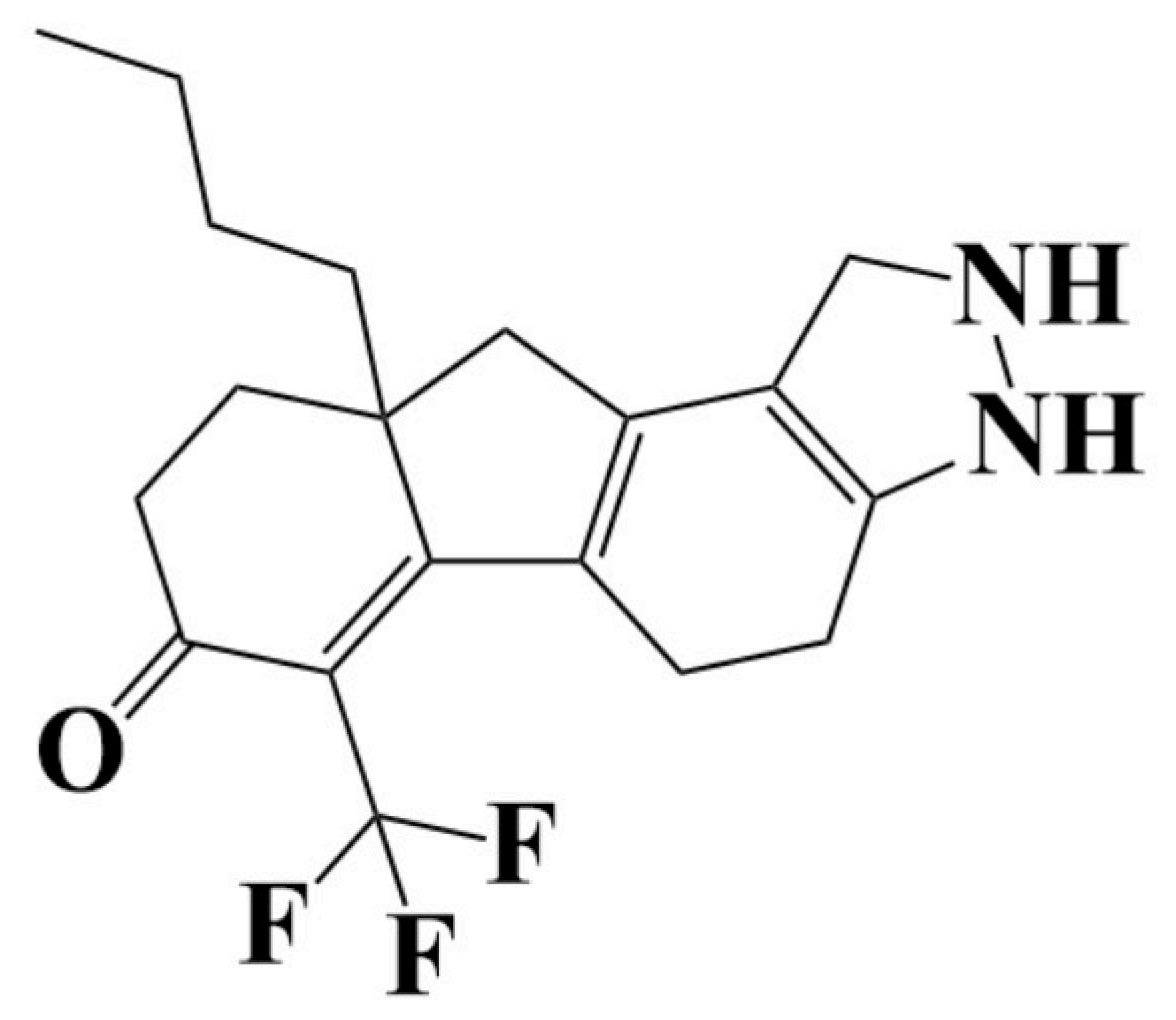	9a-butyl-6-(trifluoromethyl)-2,3,4,5,8,9,9a,10-octahydroindeno[2,1-e]indazol-7(1H)-one	−10.7 kcal/mol
Hit-2	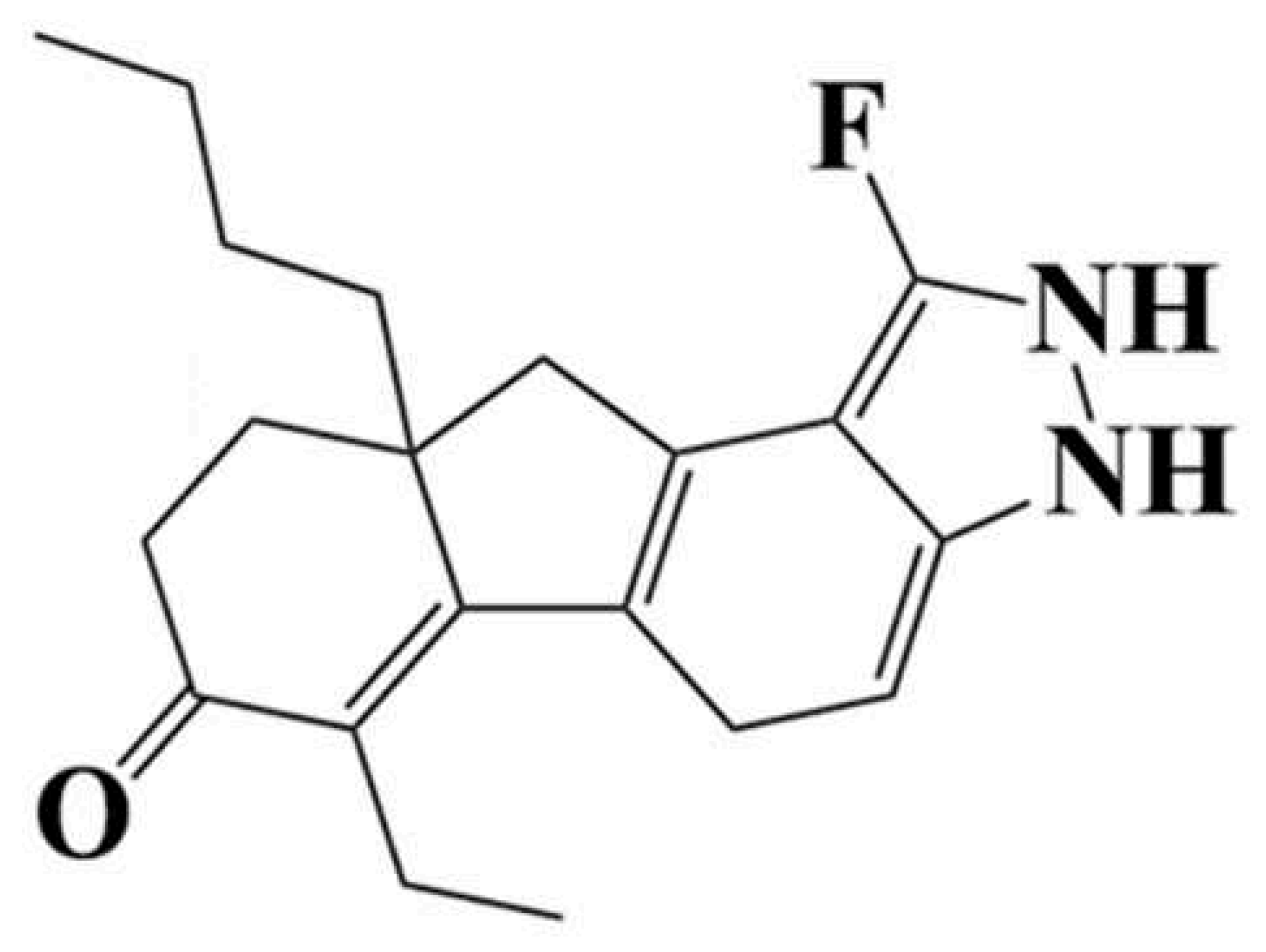	9a-butyl-6-ethyl-1-fluoro-2,3,8,9,9a,10-hexahydroindeno[2,1-e]indazol-7(5H)-one	−10.5 kcal/mol
Hit-3	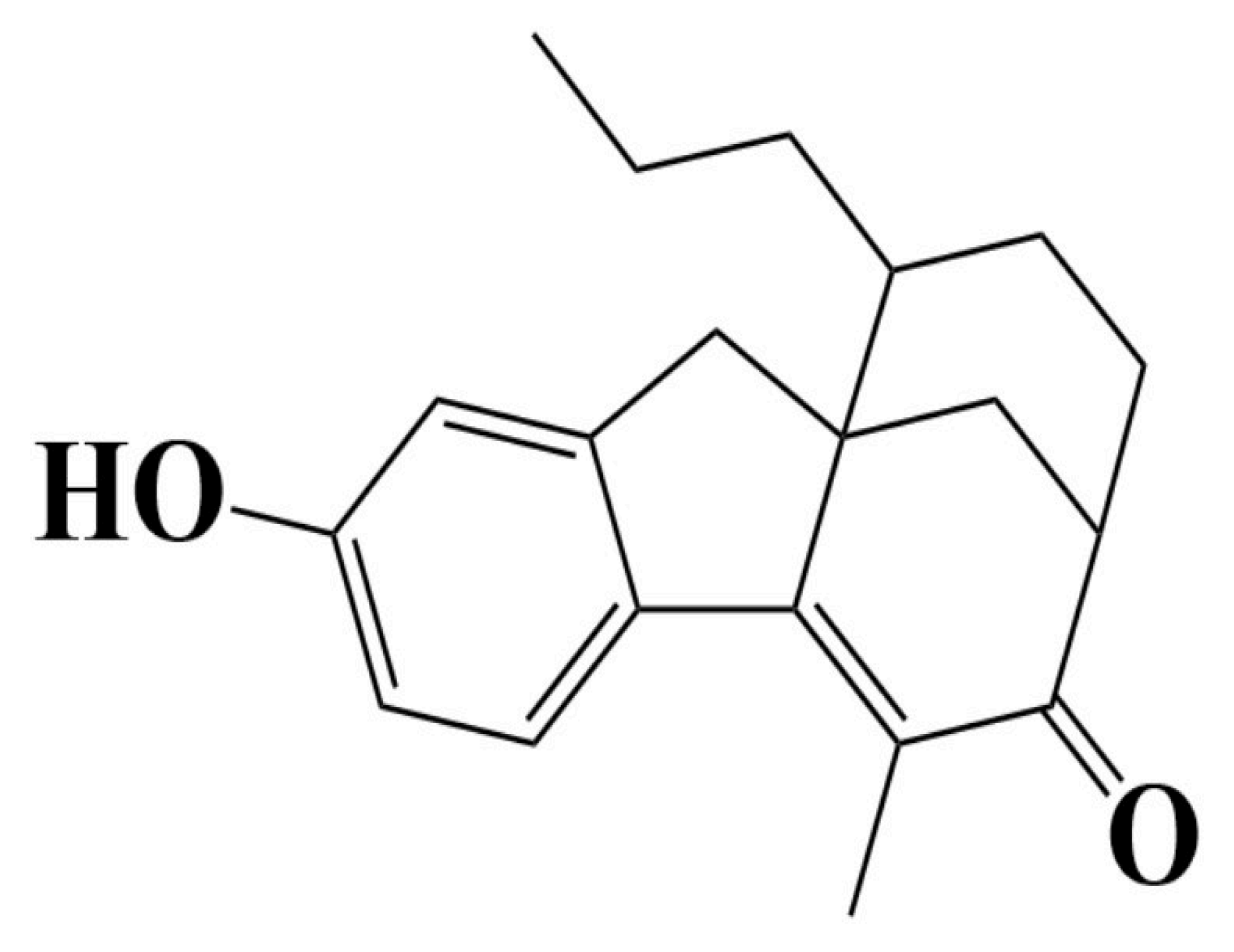	2-hydroxy-5-methyl-10-propyl-7,8,9,10-tetrahydro-7,10a-methanocycloocta[a]inden-6(11H)-one	−10.4 kcal/mol
Control	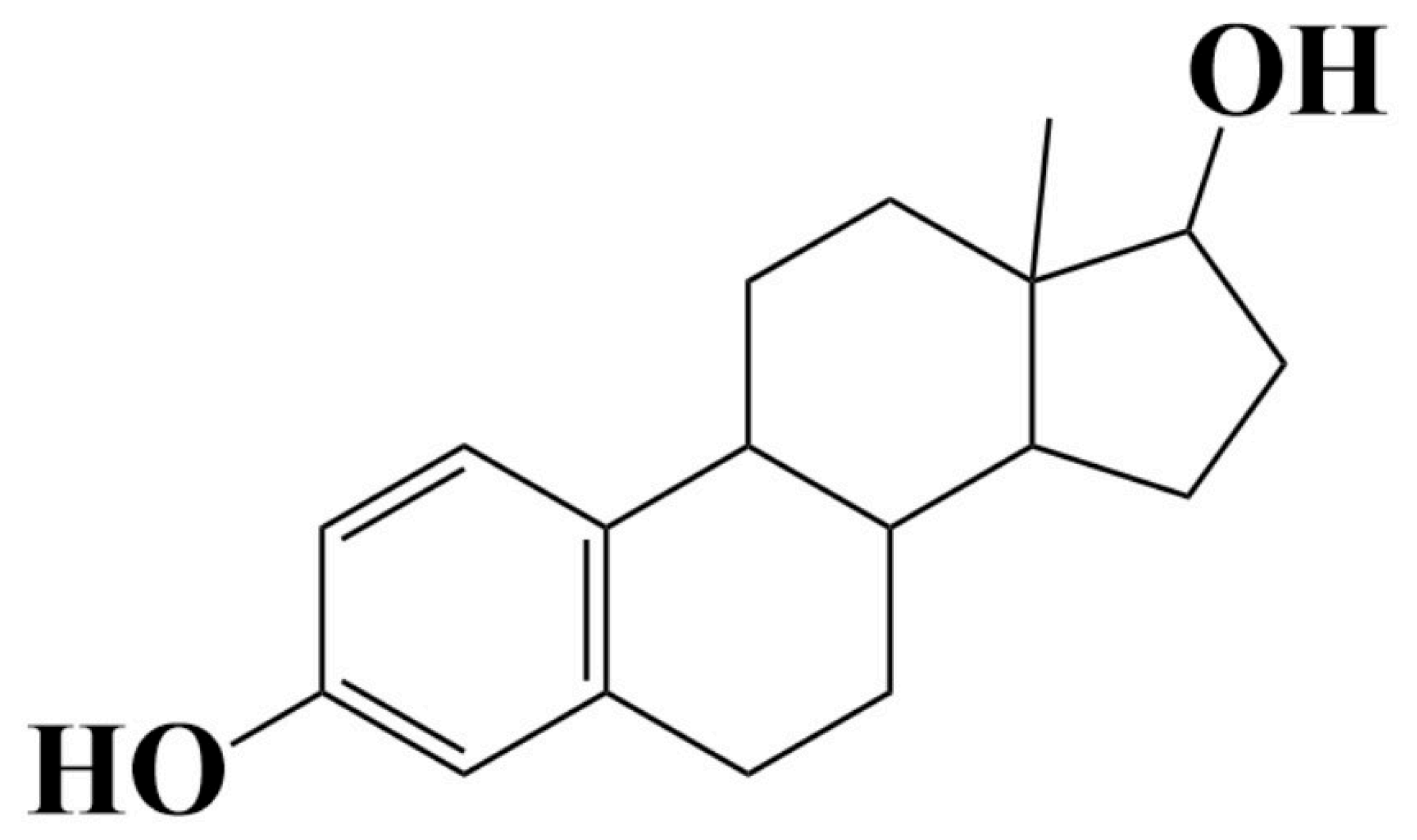	13-methyl-7,8,9,11,12,13,14,15,16,17-decahydro-6H-cyclopenta[a]phenanthrene-3,17-diol	−9.6 kcal/mol

**Table 2:** The table shows the binding free energy for the three hit compounds. The values given in the table are approximate comparative estimates and not absolute experimental data. The energy values are provided in kcal/mol.

Technique	Energy Section	Hit-1	Hit-2	Hit-3	Control
MMPBSA	Van der Waals Energy (kcal/mol)	−114.66 (±7.85)	−106.47 (±6.85)	−110.36 (±7.01)	−100.56 (±6.54)
	Electrostatic Energy (kcal/mol)	−40.21 (±4.12)	−38.69 (±4.01)	−37.49 (±4.06)	−36.40 (±3.94)
	Polar Solvation Energy (SE) (kcal/mol)	32.30 (±2.52)	35.64 (±2.97)	40.33 (±3.78)	41.06 (±4.69)
	Non-Polar SE (kcal/mol)	−7.02 (±1.20)	−6.34 (±1.36)	−6.87 (±2.05)	−5.09 (±1.03)
	Gas Phase Energy (kcal/mol)	−154.87 (±8.14)	−145.16 (±8.56)	−147.85 (±8.33)	−136.96 (±8.46)
	Total (kcal/mol)	−129.59 (±7.63)	−115.86 (±6.54)	−114.39 (±5.98)	−100.99 (±5.43)
MMGBSA	Van der Waals Energy (kcal/mol)	−114.66 (±7.85)	−106.47 (±6.85)	−110.36 (±7.01)	−100.56 (±6.54)
	Electrostatic Energy (kcal/mol)	−40.21 (±4.12)	−38.69 (±4.01)	−37.49 (±4.06)	−36.40 (±3.94)
	Polar Solvation Energy (SE) (kcal/mol)	34.52 (±4.20)	38.13 (±3.68)	39.46 (±3.45)	41.26 (±3.97)
	Non-Polar SE (kcal/mol)	−8.60 (±1.56)	−8.96 (±1.63)	−7.16 (±1.84)	−5.63 (±1.35)
	Gas Phase Energy (kcal/mol)	−154.87 (±8.14)	−145.16 (±8.56)	−147.85 (±8.33)	−136.96 (±8.46)
	Total (kcal/mol)	−128.95 (±7.41)	−115.99 (±6.48)	−115.55 (±6.66)	−101.33 (±5.37)

## Data Availability

The data generated in the work is either presented in the main manuscript and Supplementary File.

## Supplementary Materials

The following supporting information can be downloaded at: https://www.mdpi.com/article/10.3390/ph19091471/s1. Figure S1: Molecular docking pose of the control with the target protein; Figure S2: Ligand stability and solvent-accessible surface area for hits and control molecules. (A) Ligand RMSD profiles and corresponding RMSD distributions for the control and Hit-1, Hit-2, and Hit-3 complexes. (B) SASA profiles and their corresponding distributions. Dashed horizontal lines indicate the mean values; Figure S3: The frequency distributions of salt bridges are shown for (A) Hit-1, (B) Hit-2, (C) Hit-3, and (D) the control complex. The x-axis represents the number of salt bridges detected at each trajectory frame, while the y-axis indicates the corresponding frequency of frames; see Table S1. ADME assessment of three compounds and control.

## References

[1] Clusan L., Ferrière F., Flouriot G., Pakdel F. (2023). A Basic Review on Estrogen Receptor Signaling Pathways in Breast Cancer. Int. J. Mol. Sci..

[2] Strillacci A., Sansone P., Rajasekhar V.K., Turkekul M., Boyko V., Meng F., Houck-Loomis B., Brown D., Berger M.F., Hendrickson R.C. (2022). ERα-LBD, an Isoform of Estrogen Receptor Alpha, Promotes Breast Cancer Proliferation and Endocrine Resistance. npj Breast Cancer.

[3] Momenimovahed Z., Shahabinia Z., Allahqoli L., Salehiniya H. (2026). Modifiable and Nonmodifiable Risk Factors for Breast Cancer: A Comprehensive Scoping Review. Int. J. Breast Cancer.

[4] Popa M.-T., Noditi A., Peleaşa T.M., Stoleru S., Blidaru A. (2025). Breast Cancer: A Heterogeneous Pathology. Prognostic and Predictive Factors—A Narrative Review. Chirurgia.

[5] Mir M.A., Qayoom H. (2023). Introduction to Breast Cancer. Therapeutic Potential of Cell Cycle Kinases in Breast Cancer.

[6] Makhlouf S., Althobiti M., Toss M., Muftah A.A., Mongan N.P., Lee A.H.S., Green A.R., Rakha E.A. (2023). The Clinical and Biological Significance of Estrogen Receptor-Low Positive Breast Cancer. Mod. Pathol..

[7] Saha T., Lukong K.E.E. (2025). Decoding Estrogen Receptor and GPER Biology: Structural Insights and Therapeutic Advances in ERα− Positive Breast Cancer. Front. Oncol..

[8] Miziak P., Baran M., Błaszczak E., Przybyszewska-Podstawka A., Kałafut J., Smok-Kalwat J., Dmoszyńska-Graniczka M., Kiełbus M., Stepulak A. (2023). Estrogen Receptor Signaling in Breast Cancer. Cancers.

[9] Toker P., Ayten H., Demiralay Ö.D., Bınarcı B., Turan G., Olgun Ç.E., Yaşar P., Akman H.B., Muyan M. (2025). A Reappraisal of Cell Cycle Phase Enrichment in Synchronized Estrogen Receptor-Positive Cell Models Derived from Breast Adenocarcinomas. Sci. Rep..

[10] Carausu M., Bidard F.-C., Callens C., Melaabi S., Jeannot E., Pierga J.-Y., Cabel L. (2019). ESR1 Mutations: A New Biomarker in Breast Cancer. Expert Rev. Mol. Diagn..

[11] Allaoui M., Belhaouari S.B., Hedjam R., Bouanane K., Kherfi M.L. (2025). T-SNE-PSO: Optimizing t-SNE Using Particle Swarm Optimization. Expert Syst. Appl..

[12] De P., Kar S., Ambure P., Roy K. (2022). Prediction Reliability of QSAR Models: An Overview of Various Validation Tools. Arch. Toxicol..

[13] Aronskyy I., Masoudi-Sobhanzadeh Y., Cappuccio A., Zaslavsky E. (2021). Advances in the Computational Landscape for Repurposed Drugs against COVID-19. Drug Discov. Today.

[14] Roth J.P. (2026). Chemically Interpretable Explanations for Molecular Property Prediction via Fragment-Level Shapley Values. J. Chem. Inf. Model..

[15] Bernal L., Rastelli G., Pinzi L. (2025). Improving Machine Learning Classification Predictions through SHAP and Features Analysis Interpretation. J. Chem. Inf. Model..

[16] Pappalardo M., Sipala F.M., Nicolosi M.C., Guccione S., Ronsisvalle S. (2024). Recent Applications of In Silico Approaches for Studying Receptor Mutations Associated with Human Pathologies. Molecules.

[17] Khan K., Ahmad S., Aljasir M.A., Nadeem S., Siddique F., Saira, Rehman B. (2025). From Quantum Chemistry to Dynamics: Exploring Potential Inhibitors for Alongshan Virus RdRp Through Advanced Computational Techniques. J. Comput. Biophys. Chem..

[18] Maximov P.Y., Abderrahman B., Fanning S.W., Sengupta S., Fan P., Curpan R.F., Rincon D.M.Q., Greenland J.A., Rajan S.S., Greene G.L. (2018). Endoxifen, 4-Hydroxytamoxifen and an Estrogenic Derivative Modulate Estrogen Receptor Complex Mediated Apoptosis in Breast Cancer. Mol. Pharmacol..

[19] Al-Harbi A.I., Amin S., Khan K., Ullah A., Aman K., Sanami S., Alshabrmi F.M., Aljasir M.A., Siddique F., Ahmad S. (2025). Targeting Glycine N-Methyltransferase: A Computational Pipeline for Novel Anti-Pancreatic Cancer Leads. J. Comput. Biophys. Chem..

[20] Giuliani A. (2017). The Application of Principal Component Analysis to Drug Discovery and Biomedical Data. Drug Discov. Today.

[21] Graeff G.R., Tonietto Mangini A., Dorn M. (2026). Exploring Data Visualization Techniques in Molecular Dynamics: Analysis of Dynamic Cross-Correlation Maps as Dynamic Networks. Inf. Vis..

[22] Sharma R.V., Maity S. (2026). Radial Distribution Function Assisted Small Angle X-Ray Scattering Analyses towards Decoding Nano-Macromolecular Structure of Coking Coal. J. Mol. Struct..

[23] Wang Y., Zhang Y., Yu M., Xiu P., Jia Y., Chen H., Le S., Qian J., Yan J. (2025). Salt-Bridge Mediated Cooperativity and Mechanical Stabilization of Tandem Spectrin Repeats. Nanoscale Horiz..

[24] Miandad K., Ullah A., Bashir K., Khan S., Abideen S.A., Shaker B., Alharbi M., Alshammari A., Ali M., Haleem A. (2022). Virtual Screening of *Artemisia annua* Phytochemicals as Potential Inhibitors of SARS-CoV-2 Main Protease Enzyme. Molecules.

[25] Palaniappan M. (2024). Current Therapeutic Opportunities for Estrogen Receptor Mutant Breast Cancer. Biomedicines.

[26] Su J., Xin C., Shang A., Wu S., Xie Z., Xiong R., Xu X., Zhang C., Chen G., Chan Y.-T. (2025). Artificial Intelligence in Drug Discovery: A Comprehensive Review with a Case Study on Hyperuricemia, Gout Arthritis, and Hyperuricemic Nephropathy. arXiv.

[27] Bouricha E.M., Hakmi M. (2024). Investigating Lasofoxifene Efficacy against the Y537S+ F404V Double-Mutant Estrogen Receptor Alpha Using Molecular Dynamics Simulations. Bioinform. Biol. Insights.

[28] Tsou L.K., Yeh S.-H., Ueng S.-H., Chang C.-P., Song J.-S., Wu M.-H., Chang H.-F., Chen S.-R., Shih C., Chen C.-T. (2020). Comparative Study between Deep Learning and QSAR Classifications for TNBC Inhibitors and Novel GPCR Agonist Discovery. Sci. Rep..

[29] Kingston B., Pearson A., Herrera-Abreu M.T., Sim L.-X., Cutts R.J., Shah H., Moretti L., Kilburn L.S., Johnson H., Macpherson I.R. (2024). ESR1 F404 Mutations and Acquired Resistance to Fulvestrant in ESR1-Mutant Breast Cancer. Cancer Discov..

[30] Sadybekov A.V., Katritch V. (2023). Computational Approaches Streamlining Drug Discovery. Nature.

[31] Tran-Nguyen V.-K., Junaid M., Simeon S., Ballester P.J. (2023). A Practical Guide to Machine-Learning Scoring for Structure-Based Virtual Screening. Nat. Protoc..

[32] de Azevedo D.Q., Castilho R.O., Gómez-García A., Medina-Franco J.L. (2025). Molecular Databases. Computer-Aided and Machine Learning-Driven Drug Design: From Theory to Applications.

[33] Sahu S., Anmol A., Nishad T., Jujjavarapu S.E. (2026). Unveiling Molecular Signatures for Precision Drug Design: Machine Learning Insights from Trypanothione Reductase, PKC-θ, and CB1. Mol. Divers..

[34] Priya S., Tripathi G., Singh D.B., Jain P., Kumar A. (2022). Machine Learning Approaches and Their Applications in Drug Discovery and Design. Chem. Biol. Drug Des..

[35] Roy S., Chatterjee S., Bhattacharjee A., Chattopadhyay P., Saha B., Dutta S., Basu S. (2021). Overexpression of Efflux Pumps, Mutations in the Pumps’ Regulators, Chromosomal Mutations, and AAC (6′)-Ib-Cr Are Associated with Fluoroquinolone Resistance in Diverse Sequence Types of Neonatal Septicaemic *Acinetobacter baumannii*: A 7-Year Single Center. Front. Microbiol..

[36] Shahab M., Al-Madhagi H., Zheng G., Zeb A., Alasmari A.F., Alharbi M., Alasmari F., Khan M.Q., Khan M., Wadood A. (2023). Structure Based Virtual Screening and Molecular Simulation Study of FDA-Approved Drugs to Inhibit Human HDAC6 and VISTA as Dual Cancer Immunotherapy. Sci. Rep..

[37] Valencia E., Galvis M., Nisperuza J., Ballesteros V., Mesa F. (2024). In Silico Evaluation of Potential NDM-1 Inhibitors: An Integrated Docking and Molecular Dynamics Approach. Pharmaceuticals.

[38] Abdullahi M., Adeniji S.E. (2020). In-Silico Molecular Docking and ADME/Pharmacokinetic Prediction Studies of Some Novel Carboxamide Derivatives as Anti-Tubercular Agents. Chem. Afr..

[39] Amera G.M., Khan R.J., Pathak A., Jha R.K., Muthukumaran J., Singh A.K. (2020). Screening of Promising Molecules against MurG as Drug Target in Multi-Drug-Resistant-*Acinetobacter baumannii*-Insights from Comparative Protein Modeling, Molecular Docking and Molecular Dynamics Simulation. J. Biomol. Struct. Dyn..

[40] Srivastava V., Yadav A., Sarkar P. (2022). Molecular Docking and ADMET Study of Bioactive Compounds of *Glycyrrhiza glabra* against Main Protease of SARS-CoV2. Mater. Today Proc..

[41] Khare S., Chatterjee T., Gupta S., Ashish P. (2023). Bioavailability Predictions, Pharmacokinetics and Drug-Likeness of Bioactive Compounds from Andrographis Paniculata Using Swiss ADME. MGM J. Med. Sci..

[42] Sucharitha P., Reddy K.R., Satyanarayana S.V., Garg T. (2022). Absorption, Distribution, Metabolism, Excretion, and Toxicity Assessment of Drugs Using Computational Tools. Computational Approaches for Novel Therapeutic and Diagnostic Designing to Mitigate SARS-CoV-2 Infection.

[43] Patil P.B., Kumbhar S.T. (2023). Identification of Potential CDK 8 Inhibitor from Pyrimidine Derivatives via In-Silico Approach. J. Med. Pharm. Allied Sci..

[44] Akash S., Bayıl I., Rahman M.A., Mukerjee N., Maitra S., Islam M.R., Rajkhowa S., Ghosh A., Al-Hussain S.A., Zaki M.E.A. (2023). Target Specific Inhibition of West Nile Virus Envelope Glycoprotein and Methyltransferase Using Phytocompounds: An in Silico Strategy Leveraging Molecular Docking and Dynamics Simulation. Front. Microbiol..

[45] Ghorayshian A., Danesh M., Mostashari-Rad T., Fassihi A. (2023). Discovery of Novel RARα Agonists Using Pharmacophore-Based Virtual Screening, Molecular Docking, and Molecular Dynamics Simulation Studies. PLoS ONE.

[46] Sardar R., Banik R., Chowdhury S., Ghosh S. (2025). Hydrophobicity-Directed Structural Alteration in Cytochrome C Induced by Bile Salts: Physicochemical, Spectroscopic, and Atomic Force Microscopic Studies with Molecular Docking Analysis. New J. Chem..

[47] Xu Y., Guo X., Yan D., Dang X., Guo L., Jia T., Wang Q. (2023). Molecular Dynamics Simulation-Driven Focused Virtual Screening and Experimental Validation of Inhibitors for MTDH-SND1 Protein–Protein Interaction. J. Chem. Inf. Model..

[48] Rafique A. (2023). Prediction of R-134a and DNAN Physical and Transport Properties from Computer Simulations.

[49] Protti Í.F., Rodrigues D.R., Fonseca S.K., Alves R.J., de Oliveira R.B., Maltarollo V.G. (2021). Do Drug-Likeness Rules Apply to Oral Prodrugs?. ChemMedChem.

[50] Dalal V., Dhankhar P., Singh V., Singh V., Rakhaminov G., Golemi-Kotra D., Kumar P. (2021). Structure-Based Identification of Potential Drugs Against FmtA of Staphylococcus Aureus: Virtual Screening, Molecular Dynamics, MM-GBSA, and QM/MM. Protein J..

[51] Kumari M., Singh R., Subbarao N. (2022). Exploring the Interaction Mechanism between Potential Inhibitor and Multi-Target Mur Enzymes of *Mycobacterium tuberculosis* Using Molecular Docking, Molecular Dynamics Simulation, Principal Component Analysis, Free Energy Landscape, Dynamic Cross-Correlati. J. Biomol. Struct. Dyn..

[52] Ahmad S., Raza S., Uddin R., Azam S.S. (2017). Binding Mode Analysis, Dynamic Simulation and Binding Free Energy Calculations of the MurF Ligase from *Acinetobacter baumannii*. J. Mol. Graph. Model..

