# Supplementary material for: Cracking ERα Y537S Resistance: Explainable Machine Learning-Guided Discovery and Molecular Dynamics Validation of Stable Candidate Ligands
Source: Pharmaceuticals (Basel). 2026 Sep 16;19(9):1471. doi: 10.3390/ph19091471 (PMC13609935; doi:10.3390/ph19091471)
Supplement: Supplementary file 1 [file pharmaceuticals-19-01471-s001.zip › pharmaceuticals-4480550-supplementary.pdf]

Supplementary File

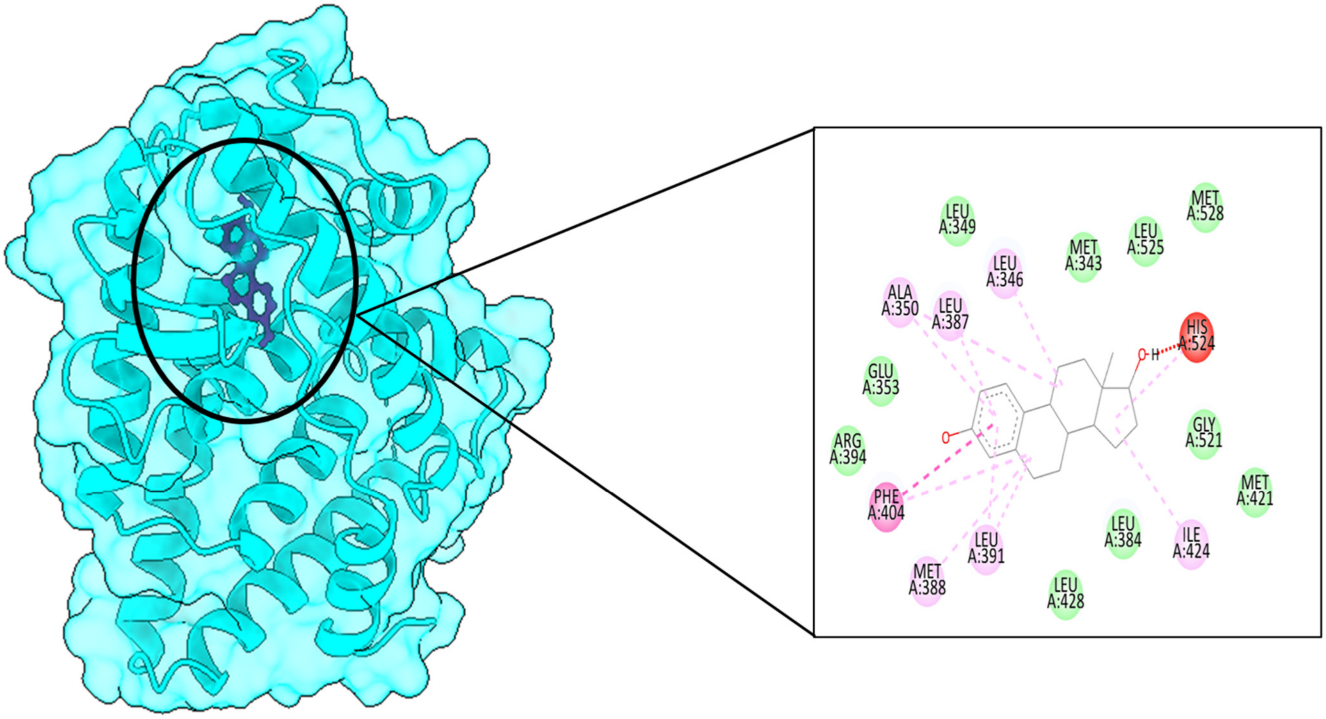

Figure S1: Molecular docking pose of the control with the target protein.

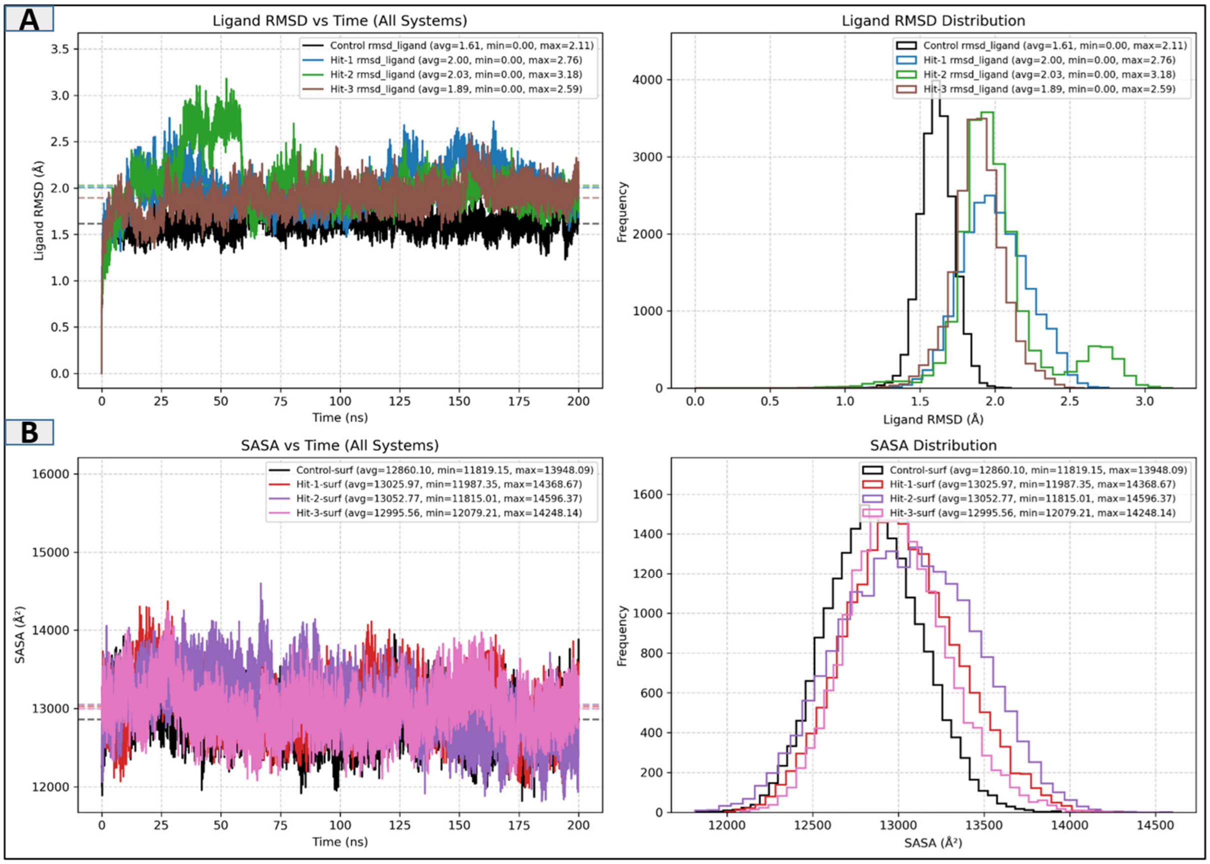

Figure S2: Ligand stability and solvent-accessible surface area for hits and control molecules. (A) Ligand RMSD profiles and corresponding RMSD distributions for the control and Hit-1, Hit-2, and Hit-3 complexes. (B) SASA profiles and their corresponding distributions. Dashed horizontal lines indicate the mean values.

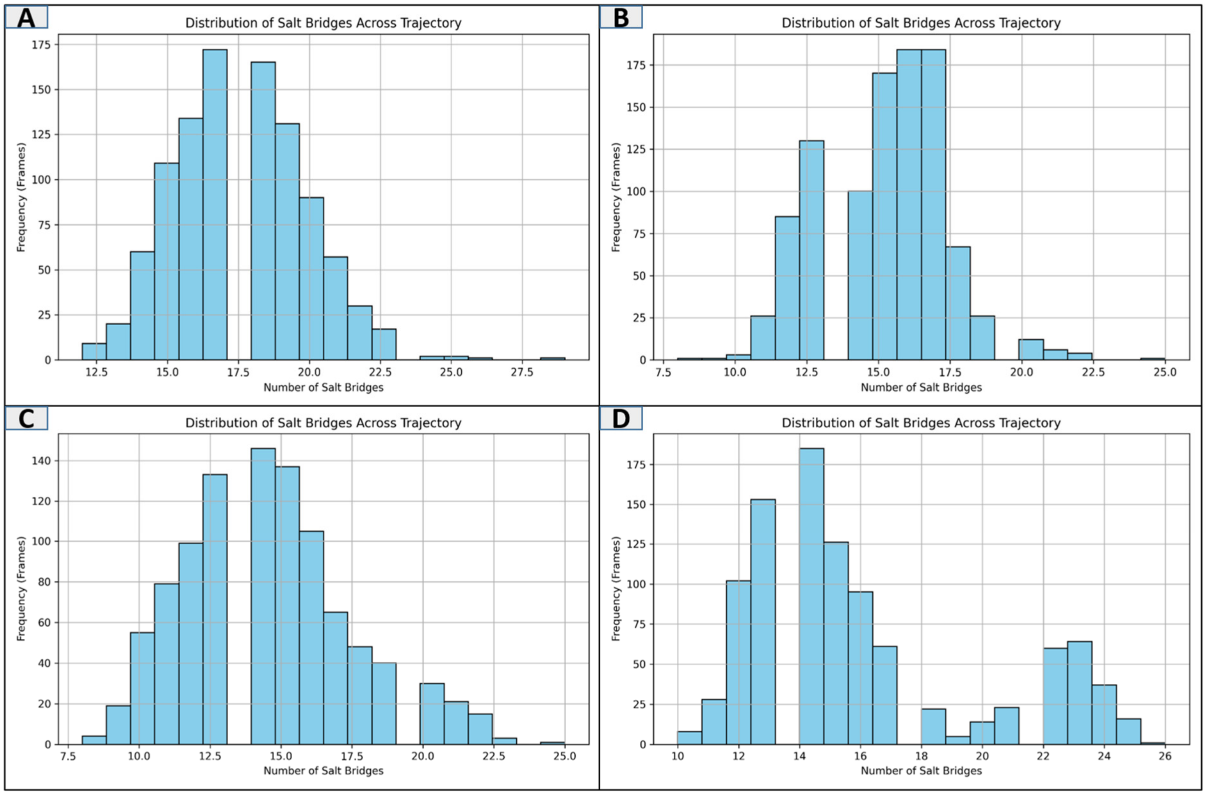

Figure S3: The frequency distributions of salt bridges are shown for (A) Hit-1, (B) Hit-2, (C) Hit-3, and (D) the control complex**.** The x-axis represents the number of salt bridges detected at each trajectory frame, while the y-axis indicates the corresponding frequency of frames.

Table S1. ADME assessment of three compounds and Control.

**Hit-1**	
SMILES	CCCCC12CCC(=O)C(=C2C2=C(C1)C1=C(CC2)NNC1)C(F)(F)F
**Physicochemical Properties**	
Formula	C19H23F3N2O
Molecular weight	352.39 g/mol
Num. heavy atoms	25
Num. arom. heavy atoms	0
Fraction Csp3	0.63
Num. rotatable bonds	4
Num. H-bond acceptors	5
Num. H-bond donors	2
Molar Refractivity	97.13
TPSA	41.13 Å²
**Lipophilicity**	
Log Po/w (iLOGP)	Temporarily unavailable
Log Po/w (XLOGP3)	2.9

Log Po/w (WLOGP)	4.74
Log Po/w (MLOGP)	3.68
Log Po/w (SILICOS-IT)	4.76
Consensus Log Po/w	4.02
**Water Solubility**	
Log S (ESOL)	-3.59
Solubility	9.10e-02 mg/ml ; 2.58e-04 mol/l
Class	Soluble
Log S (Ali)	-3.42
Solubility	1.33e-01 mg/ml ; 3.76e-04 mol/l
Class	Soluble
Log S (SILICOS-IT)	-6.1
Solubility	2.82e-04 mg/ml ; 8.00e-07 mol/l
Class	Poorly soluble
**Pharmacokinetics**	
GI absorption	High
BBB permeant	Yes
P-gp substrate	Yes
CYP1A2 inhibitor	Temporarily unavailable
CYP2C19 inhibitor	Temporarily unavailable
CYP2C9 inhibitor	Temporarily unavailable
CYP2D6 inhibitor	Temporarily unavailable
CYP3A4 inhibitor	Temporarily unavailable
Log Kp (skin permeation)	-6.39 cm/s
**Druglikeness**	
Lipinski	Yes; 0 violation
Ghose	Yes
Veber	Yes
Egan	Yes
Muegge	Yes
Bioavailability Score	0.55
**Medicinal Chemistry**	
PAINS	0 alert
Brenk	0 alert
Leadlikeness	No; 1 violation: MW>350
Synthetic accessibility	4.76
**Hit-2**	
SMILES	CCCCC12CCC(=O)C(=C2C2=C(C1)c1c(F)[nH][nH]c1=CC2)CC
**Physicochemical Properties**	
Formula	C20H25FN2O
Molecular weight	328.42 g/mol

Num. heavy atoms	24
Num. arom. heavy atoms	5
Fraction Csp3	0.55
Num. rotatable bonds	4
Num. H-bond acceptors	2
Num. H-bond donors	2
Molar Refractivity	95.17
TPSA	48.65 Å²
**Lipophilicity**	
Log Po/w (iLOGP)	Temporarily unavailable
Log Po/w (XLOGP3)	4.61
Log Po/w (WLOGP)	4.77
Log Po/w (MLOGP)	3.74
Log Po/w (SILICOS-IT)	3.97
Consensus Log Po/w	4.27
**Water Solubility**	
Log S (ESOL)	-4.67
Solubility	7.01e-03 mg/ml ; 2.13e-05 mol/l
Class	Moderately soluble
Log S (Ali)	-5.36
Solubility	1.44e-03 mg/ml ; 4.40e-06 mol/l
Class	Moderately soluble
Log S (SILICOS-IT)	-5.62
Solubility	7.91e-04 mg/ml ; 2.41e-06 mol/l
Class	Moderately soluble
**Pharmacokinetics**	
GI absorption	High
BBB permeant	Yes
P-gp substrate	No
CYP1A2 inhibitor	Temporarily unavailable
CYP2C19 inhibitor	Temporarily unavailable
CYP2C9 inhibitor	Temporarily unavailable
CYP2D6 inhibitor	Temporarily unavailable
CYP3A4 inhibitor	Temporarily unavailable
Log Kp (skin permeation)	-5.03 cm/s
**Druglikeness**	
Lipinski	Yes; 0 violation
Ghose	Yes
Veber	Yes
Egan	Yes
Muegge	Yes
Bioavailability Score	0.55
**Medicinal Chemistry**	

PAINS	0 alert
Brenk	0 alert
Leadlikeness	No; 1 violation: XLOGP3>3.5
Synthetic accessibility	4.66
**Hit-3**	
SMILES	CCCC1CCC2CC31Cc1c(C3=C(C2=O)C)ccc(c1)O
**Physicochemical Properties**	
Formula	$C_{20}H_{24}O_2$
Molecular weight	296.40 g/mol
Num. heavy atoms	22
Num. arom. heavy atoms	6
Fraction Csp3	0.55
Num. rotatable bonds	2
Num. H-bond acceptors	2
Num. H-bond donors	1
Molar Refractivity	90
TPSA	37.30 Å^2
**Lipophilicity**	
Log Po/w (iLOGP)	Temporarily unavailable
Log Po/w (XLOGP3)	4.51
Log Po/w (WLOGP)	4.51
Log Po/w (MLOGP)	3.82
Log Po/w (SILICOS-IT)	4.82
Consensus Log Po/w	4.41
**Water Solubility**	
Log S (ESOL)	-4.59
Solubility	7.64e-03 mg/ml ; 2.58e-05 mol/l
Class	Moderately soluble
Log S (Ali)	-5.01
Solubility	2.87e-03 mg/ml ; 9.67e-06 mol/l
Class	Moderately soluble
Log S (SILICOS-IT)	-5.55
Solubility	8.32e-04 mg/ml ; 2.81e-06 mol/l
Class	Moderately soluble
**Pharmacokinetics**	
GI absorption	High
BBB permeant	Yes
P-gp substrate	No
CYP1A2 inhibitor	Temporarily unavailable
CYP2C19 inhibitor	Temporarily unavailable
CYP2C9 inhibitor	Temporarily unavailable
CYP2D6 inhibitor	Temporarily unavailable

CYP3A4 inhibitor	Temporarily unavailable
Log Kp (skin permeation)	-4.91 cm/s
**Druglikeness**	
Lipinski	Yes; 0 violation
Ghose	Yes
Veber	Yes
Egan	Yes
Muegge	Yes
Bioavailability Score	0.55
**Medicinal Chemistry**	
PAINS	0 alert
Brenk	0 alert
Leadlikeness	No; 1 violation: XLOGP3>3.5
Synthetic accessibility	5.01
**Control**	
SMILES	Oc1ccc2c(c1)CCC1C2CCC2(C1CCC2O)C
**Physicochemical Properties**	
Formula	C18H24O2
Molecular weight	272.38 g/mol
Num. heavy atoms	20
Num. arom. heavy atoms	6
Fraction Csp3	0.67
Num. rotatable bonds	0
Num. H-bond acceptors	2
Num. H-bond donors	2
Molar Refractivity	81.03
TPSA	40.46 Å²
**Lipophilicity**	
Log Po/w (iLOGP)	Temporarily unavailable
Log Po/w (XLOGP3)	4.01
Log Po/w (WLOGP)	3.61
Log Po/w (MLOGP)	3.53
Log Po/w (SILICOS-IT)	3.25
Consensus Log Po/w	3.6
**Water Solubility**	
Log S (ESOL)	-4.28
Solubility	1.44e-02 mg/ml ; 5.28e-05 mol/l
Class	Moderately soluble
Log S (Ali)	-4.56
Solubility	7.47e-03 mg/ml ; 2.74e-05 mol/l
Class	Moderately soluble

Log S (SILICOS-IT)	-3.75
Solubility	4.85e-02 mg/ml ; 1.78e-04 mol/l
Class	Soluble
**Pharmacokinetics**	
GI absorption	High
BBB permeant	Yes
P-gp substrate	Yes
CYP1A2 inhibitor	Temporarily unavailable
CYP2C19 inhibitor	Temporarily unavailable
CYP2C9 inhibitor	Temporarily unavailable
CYP2D6 inhibitor	Temporarily unavailable
CYP3A4 inhibitor	Temporarily unavailable
Log Kp (skin permeation)	-5.11 cm/s
**Druglikeness**	
Lipinski	Yes; 0 violation
Ghose	Yes
Veber	Yes
Egan	Yes
Muegge	Yes
Bioavailability Score	0.55
**Medicinal Chemistry**	
PAINS	0 alert
Brenk	0 alert
Leadlikeness	No; 1 violation: XLOGP3>3.5
Synthetic accessibility	3.49
